# Comparison of the adolescent and adult mouse prefrontal cortex proteome

**DOI:** 10.1371/journal.pone.0178391

**Published:** 2017-06-01

**Authors:** Abigail E. Agoglia, Sarah E. Holstein, Amanda T. Small, Marina Spanos, Brainard M. Burrus, Clyde W. Hodge

**Affiliations:** 1 Bowles Center for Alcohol Studies, University of North Carolina at Chapel Hill, Chapel Hill, North Carolina, United States of America; 2 Curriculum in Neurobiology, University of North Carolina at Chapel Hill, Chapel Hill, North Carolina, United States of America; 3 Department of Psychiatry, University of North Carolina at Chapel Hill, Chapel Hill, North Carolina, United States of America; Technion Israel Institute of Technology, ISRAEL

## Abstract

Adolescence is a developmental period characterized by unique behavioral phenotypes (increased novelty seeking, risk taking, sociability and impulsivity) and increased risk for destructive behaviors, impaired decision making and psychiatric illness. Adaptive and maladaptive adolescent traits have been associated with development of the medial prefrontal cortex (mPFC), a brain region that mediates regulatory control of behavior. However, the molecular changes that underlie brain development and behavioral vulnerability have not been fully characterized. Using high-throughput 2D DIGE spot profiling with identification by MALDI-TOF mass spectrometry, we identified 62 spots in the PFC that exhibited age-dependent differences in expression. Identified proteins were associated with diverse cellular functions, including intracellular signaling, synaptic plasticity, cellular organization and metabolism. Separate Western blot analyses confirmed age-related changes in DPYSL2, DNM1, STXBP1 and CFL1 in the mPFC and expanded these findings to the dorsal striatum, nucleus accumbens, motor cortex, amygdala and ventral tegmental area. Ingenuity Pathway Analysis (IPA) identified functional interaction networks enriched with proteins identified in the proteomics screen, linking age-related alterations in protein expression to cellular assembly and development, cell signaling and behavior, and psychiatric illness. These results provide insight into potential molecular components of adolescent cortical development, implicating structural processes that begin during embryonic development as well as plastic adaptations in signaling that may work in concert to bring the cortex, and other brain regions, into maturity.

## Introduction

Adolescence is a critical developmental period during which organisms make the transition from childhood to adulthood. This time period is characterized by significant changes in brain architecture [1], pubertal development and sexual maturity, and several unique behavioral characteristics, including increases in risk-taking, sociability, novelty-seeking, reward sensitivity and impulsivity [2]. Both the physical and behavioral manifestations of adolescence are conserved across mammalian species, facilitating the use of rodent models in the study of adolescent development [3]. Adolescence is also a potentially vulnerable time, associated with increased rates of alcohol and drug use, risky sexual practices, and reckless driving [4]. Additionally, adolescence in humans and rodents is an epoch of heightened stress, characterized by increases in anxiety- like behavior as well as enhanced sensitivity of the hypothalamic-pituitary-adrenal axis (HPA axis) response to stressors [5]. Importantly, adolescence is the typical time of onset of many neurological and psychiatric conditions, including epilepsy, neurodegenerative disorders and neuromuscular dysfunction [6] as well as anxiety, impulse-control, substance use, schizophrenia and mood disorders [7]. In spite of widespread recognition of the adaptive and maladaptive changes associated with adolescence, the specific neuronal mechanisms that usher the brain into adult maturity (and potentially mediate both behavior and dysfunction) remain unclear.

During adolescence, the brain undergoes substantial structural and functional alteration. Of considerable significance is the decline of cortical gray matter, which usually begins in late childhood/early adolescence [8] and may be driven by both synaptic pruning [9] and enhanced myelination of existing axons [10]. Notably, loss of gray matter density follows an anterior-to-posterior trajectory, with maturation occurring first in sensorimotor areas and last in higher-order regions such as the prefrontal cortex (PFC) [11]. The PFC is functionally involved in the executive control of behavior and decision-making processes [12], and the relative immaturity of the adolescent PFC is associated with lack of inhibitory control over behavior exhibited by adolescents [13, 14]. At the same time, limbic brain areas associated with emotional arousal and reward, such as the amygdala and nucleus accumbens, reach maturity earlier than cortical regions and receive fewer neural projections from the immature PFC [15], resulting in an imbalance in top-down control of limbic regions and greater reward seeking and impulsive behavior in adolescents [16]. The PFC is therefore both a site of significant neuronal development during adolescence and a potential contributor to adolescent behavioral phenotypes.

Previous reports have begun to characterize the development of the PFC proteome from birth to adulthood [17–20], but several important questions have not been addressed by the existing literature. The specific alterations of protein expression and network function during adolescence remain unclear, due to the combination of pre-adolescent and adolescent data for comparison with adults in prior studies. Further, the majority of findings in the developing PFC to date have focused on the synaptic fraction of proteins. Although this strategy brings important insight into the development of synaptic connections and signaling during brain development, proteins that are expressed outside of the membrane fraction may play an important role in the maturation of the adolescent cortex. Additionally, some studies have failed to distinguish between the subregions of the PFC collected for analysis. The prelimbic and infralimbic PFC have different projections and different functional roles in behavior and therefore may be subject to different developmental processes during the adolescent period [21]. Finally, previous reports have focused exclusively on the expression of proteins in the PFC, creating uncertainty as to whether the observed protein expression differences in the PFC are unique to that region or part of a general developmental trend across multiple brain regions.

To investigate the subcellular machinery involved in adolescent brain development and behavior, we used a high-throughput unbiased proteomics analysis to characterize age differences in protein expression between adolescent and adult male C57BL/6J mice. Mice have a defined period of adolescence (approximately 2 weeks) in which they display “adolescent typical” behavior, such as impulsivity and novelty seeking [22], and were therefore a useful model for these studies. We chose to focus on the transition from early- to mid-adolescence in order to capture a snapshot of the adolescent brain midway through maturation to adulthood, and therefore collected tissue on post-natal day (PND) 36 [23]. We focused on the prelimbic medial prefrontal cortex (mPFC) as this region is critically involved in executive control of behavior and sends projections to the amygdala and nucleus accumbens [24]. We also used a bioinformatics approach to identify protein networks that may play a role in adolescent brain development, particularly neurochemical signaling and structural alterations. By identifying proteins that are differentially expressed in the adult and adolescent mouse mPFC, as well as additional brain regions of interest, these experiments give insight into cellular correlates of adolescent-typical behaviors and dysfunctions.

## Materials and methods

### Subjects

All procedures were performed in accordance with the NIH Guide to the Care and Use of Laboratory Animals [25] and approved by the Internal Review Board as compliant with all institutional guidelines at the University of North Carolina, Chapel Hill (approved protocol number: 13–217).

#### Proteomic analysis

Adolescent (postnatal day 21 [PND21]) and adult (PND65 ± 3) male C57BL/6J mice (Jackson Laboratories, Bar Harbor, ME) were pair-housed upon arrival in standard laboratory cages with corn-cob bedding and a small PVC tube for environmental enrichment. Food and water were available *ad libitum* for the duration of the experiment. Subjects were minimally handled throughout the experiment to minimize stress.

#### Immunoblotting

An additional group of adolescent (PND 21) and adult (PND 65 ± 3) male C57BL/6J mice (Jackson) were housed and handled under identical conditions to the proteomics cohort in order to confirm expression changes observed in the proteomics experiments using Western blots.

### Proteomic analysis

**Fig 1:**
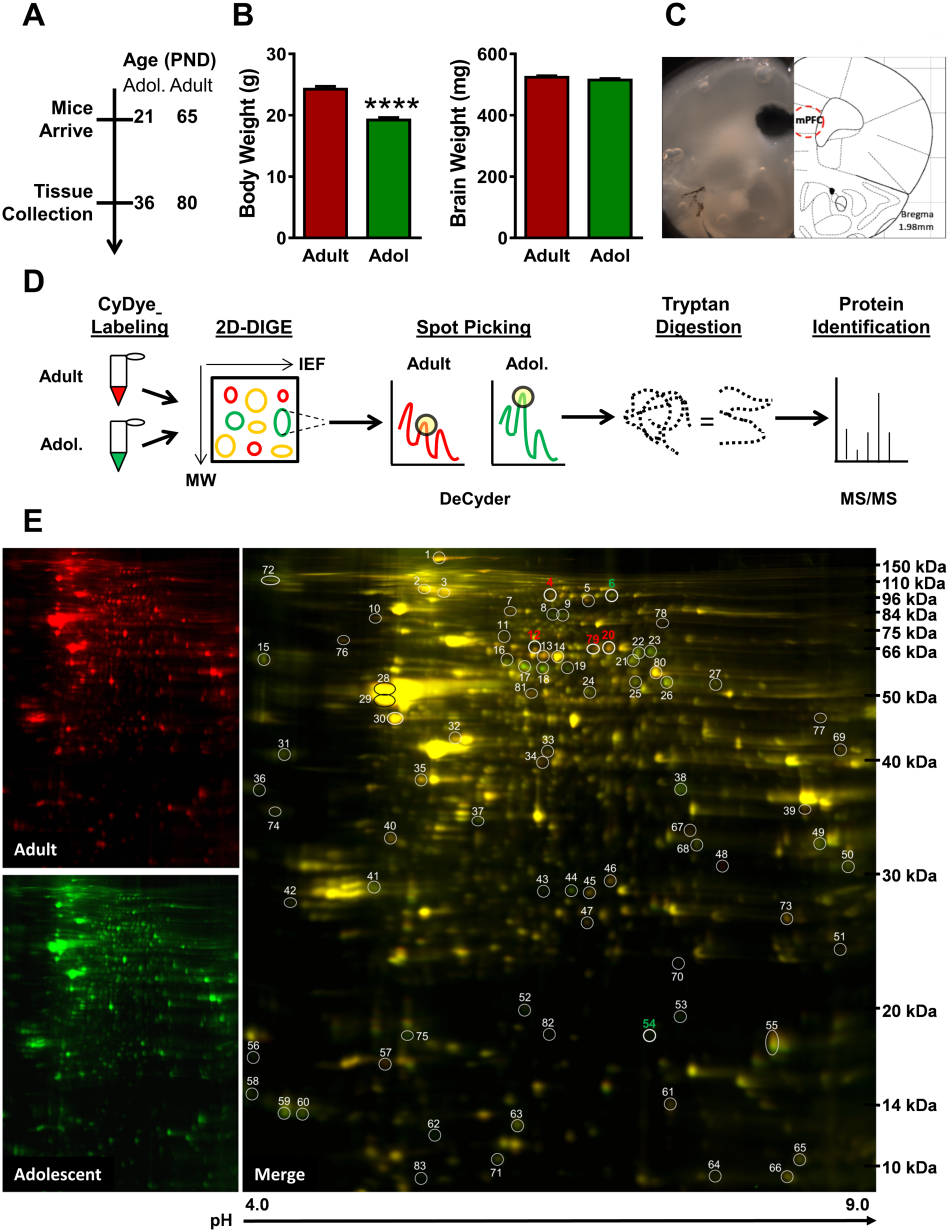
Proteomic analysis of the adolescent and adult medial prefrontal cortex (mPFC). **(A)** Timeline of experimental procedure. **(B)** Adult body weight was significantly greater than adolescent body weight at tissue collection (left), but brain weight did not differ between the two ages (right). **(C)** Photomicrograph (left) and schematic (right) of mouse brain section showing location of mPFC tissue punch for two-dimensional differential in-gel electrophoresis (2D-DIGE) and immunoblot studies. **(D)** Schematic describing proteomics workflow. Adult tissue is combined with Cy3 (red) and adolescent with Cy2 (green) dye and run in 2D-DIGE, with protein separating in the *y* plane via molecular weight and the *x* plane via isoelectrical focusing (IEF). DeCyder software identifies protein spots with significantly different florescent signals. Selected spots are subjected to tryptan digestion and identified via tandem MALDI TOF/TOF mass spectrometry. **(E)** Representative 2D-DIGE gel run in the proteomics analysis of mPFC. Adult samples were combined with red Cydye (left top); adolescent samples were combined with green CyDye (left bottom). Overlay of adult and adolescent samples (right). IEF is indicated on the *x* axis with pH values and molecular weight is indicated on the *y* axis in kDa. Circles indicate location of differentially expressed spots on the gel, with numeric markers prior to identification. Focus proteins are indicated in bold, with red representing increased expression in adults and green representing increased expression in adolescents. (**** indicates p≤0.0001).

Adolescent and adult mouse mPFC proteomes were analyzed utilizing 2-Dimentional in-gel electrophoresis (2D-DIGE) for protein expression profiling, DeCyder software for selection of significantly altered spots, and matrix-assisted laser desorption/ionization-Time of Flight (MALDI-TOF) mass spectrometry (MS) for protein identification (Fig 1D) [26].

#### Tissue collection

Fifteen days after arrival (Fig 1A), 12 adolescent (P36) and 12 adult (P80 ± 3) mice were weighed and deeply anesthetized with sodium pentobarbital (150 mg/kg, IP). Once anesthetized, mice were transcardially perfused with ice-cold phosphate-buffered saline (PBS, 0.1M, pH 7.4) for 2 min at rate of 3 ml/min in order to remove blood from the brain tissue. The brain was then quickly isolated and flash-frozen in isopentane (2-methylbutane; Sigma-Aldrich, St. Louis, MO) at -20 –-30°C for 1 min, weighed, and stored at -80°C. To isolate the mPFC, tissue was sliced coronally on a cryostat (Leica CM3050S, Leica Biosystems, Buffalo Grove, IL), with a 0.5 mm tissue slice being taken between +1.5–2.0 mm (± 0.2) anterior to Bregma. The mPFC was dissected out bilaterally using a 1.0 mm tissue punch (Fig 1C). The isolated tissue included the prelimbic and dorsal infralimbic cortices, as well as the posterior anterior cingulate cortex. Tissue was pooled from n = 3 mice per age group resulting in a final group size of *N* = 4 adolescent and *N* = 4 adult mPFC samples for analysis on 4 replicate 2D gels. Protein samples were kept at -80°C and shipped to Applied Biomics (Hayward, CA) for 2-D DIGE analysis.

#### Tissue preparation

Tissue samples were sonicated on ice in 2D lysis buffer (2 M thiourea, 7 M urea, 4% CHAPS, 30 mM Tris-HCl, pH 8.8) and shaken for 30 min at room temperature. Samples were then spun at 14000 rpm at 4°C for 30 min, and the resulting supernatant was collected. Protein concentrations were determined using the Bio-Rad protein assay method (Hercules, CA), and samples were diluted to 5 mg/ml in 2-D lysis buffer.

#### CyDye labeling

Adult and adolescent tissue samples (30 μg) were combined within age group with 1.0 μl of the appropriate diluted CyDye (Cy2, Cy3, or Cy5; 1:5 concentration, diluted with dimethylformamide (DMF) from a 1 nmol/μl stock), vortexed, and kept on ice for 30 min in the dark. Afterwards, 1.0 μl of 10 mM Lysine was added to each sample, vortexed, and incubated on ice for 15 min in the dark. The CyDye-labeled samples (Cy2, Cy3, Cy5) for each age group were then combined and mixed with a 2X 2-D sample buffer (8 M urea, 4% CHAPS, 20 mg/ml dithiothreitol, 2% pharmalytes, trace amount of bromophenol blue) and 100 μl of Destreak Solution and Rehydration Buffer (7 M urea, 2 M thiourea, 4% CHAPS, 20 mg/ml DTT, 1% pharmalytes, trace amount of bormophenol blue) to a final volume of 350 μl for the 18 cm IPG strip. Labeled samples were mixed well and spun before loading into the strip holder.

#### 2D-DIGE

Following loading of samples into the 18 cm IPG strip holder, the strip was placed facing down and 1.5 ml mineral oil was added to the top of the strip. Samples were then run using isoelectric focusing (IEF) under dark conditions at 20°C using an established protocol (GE Healthcare, Pittsburgh, PA). Following IEF, the IPG strips were incubated in fresh equilibration buffer 1 (50 mM Tris-HCl, pH 8.8, containing 6 M urea, 30% glycerol, 2% SDS, trace amount of bromophenol blue, 10 mM DTT) for 15 min with gentle shaking. Strips were then washed in fresh equilibration buffer 2 (50 mM Tris-HCl, pH 8.8, containing 6 M urea, 30% glycerol, 2% SDS, trace amount of bromophenol blue, and 45 mg/ml lodacetamide) for 10 min with gentle shaking. IPG strips were then washed and transferred to a 12% SDS gel (prepared using low fluorescent glass plates) and sealed with 0.5% w/v agarose solution in SDS-gel running buffer. Gels were run at 15°C.

#### Image scan and data analysis

Immediately following the SDS-PAGE portion of the 2D-DIGE experiment, image scans were conducted using a Typhoon TRIO imager (GEHealthcare). Scanned images were analyzed by ImageQuantTL (IQTL) software (GE Healthcare) and subjected to both in-gel and cross-gel analyses using the DeCyder software package (v. 6.5, GE Healthcare), which provided a ratio change of protein expression from the in-gel analyses.

#### Spot picking and trypsin digestion

Protein spots that met our *a priori* spot-picking criteria (differential expression in the same direction in all four gels, an overall significant difference in expression [*p*<0.05], and a 1.2-fold change or greater increase or decrease in expression) were isolated by the Ettan Spot Picker (GE Healthcare) (Fig 1D). Gel spots were washed and digested in-gel with a modified porcine trypsin protease (Trypsin Gold, Promega, Madison, WI). Digested peptides were desalted (Zip-tip C18 column, Millipore, Billerica, MA) and eluted with 0.5 μl of matrix solution (α-cyano-4-hydroxycinnamic acid, 5 mg/ml in 50% acetonitrile, 0.1% trifluoroacetic acid, 25 mM ammonium bicarbonate) and spotted on the matrix-assisted laser desorption/ionization (MALDI) plate.

#### Mass spectrometry

Both MALDI-TOF (time-of-flight) mass spectrometry (MS) and TOF/TOF (tandem MS/MS) analyses were performed on a 5800 mass spectrometer (AB Sciex, Redwood City, CA). Mass spectra from the MALDI-TOF analysis were acquired in reflectron positive ion mode (average of 2000 laser shots/spectrum), whereas the TOF/TOF tandem MS fragmentation spectra were acquired for each sample (average of 2000 laser shots/fragementation spectrum) on each of the 10 most abundant ions present in the sample (with the exclusion of trypsin autolytic peptides and other background ions).

#### Database search

Resulting peptide masses and fragmentation spectra were submitted to GPS Explorer (v. 3.5) with the MASCOT search engine (Matrix Science, Boston, MA) in order to explore the database of the National Center for Biotechnology Information non-redundant (NCBInr). Searches were not constrained by protein molecular weight or isoelectric point; additionally, the search allowed for variable carbamidomethylation of cysteine and oxidation of methionine residues, and one missed cleavage was allowed in the search parameters. Both ion score (statistical likelihood that a peptide sequence experimentally observed and identified in the MASCOT database are matched based on random chance [-Log_10_P]) and protein score (sum of the highest ion scores for each sequence) were calculated; increased protein score indicates increased confidence in the identification of the protein. Candidate proteins with a protein score confidence interval greater than 95% were considered significant. For samples with multiple candidate proteins exceeding the identification criteria, an identity was assigned based on the highest protein score. Full identification data, including all candidate proteins for each spot, are available on the open access proteomics data repository ProteomeXchange (www.proteomexchange.org).

### Bioinformatics

#### Pathway analysis

Protein identifiers, fold-change, and *p*-values from the proteomic analysis were uploaded to QIAGEN’s Ingenuity Pathway Analysis (IPA; QIAGEN Redwood City, CA) system for dataset enrichment. A Core Analysis was performed on the dataset, using the following parameters: reference set- Ingenuity Knowledge Base (genes only), relationships- direct and indirect, networks- interaction, data sources- all, confidence- experimentally observed, species- mouse, tissues and cells lines- nervous system/CNS cell lines. Proteins were assessed via Global Functional Analysis (GFA) and Global Canonical Pathways (GCP) to identify functional protein networks and canonical signaling systems that were impacted by developmental state. Statistical significance of the predicted functions and pathways was determined using the right-tailed Fisher’s Exact Test, where significance indicates that an identified set of proteins is overrepresented in a set of proteins with known function and is interpreted to indicate altered function in the experimental set.

Protein interaction networks were derived via Ingenuity’s interconnectivity algorithm. *p* values, representing the probability of finding proteins identified in the proteomics analysis (Focus Molecules) in a set of *n* genes randomly selected from the Global Molecular Network, were calculated using Fisher’s Exact Test and displayed as *p*-scores [*p*-score = -log_10_ (*p*-value); i.e. *p*-score indicates the exponent of the significance of the protein network identification]. Ingenuity Pathway Designer (QIAGEN) was used to visualize the statistically significant protein interaction networks revealed by GFA.

### Immunoblotting: mPFC confirmations and additional brain regions

#### Tissue collection

Brain tissue was collected from adolescent (P36) and adult (P80 ± 3) [n = 12/age] as described above. In addition to the mPFC as described, the dorsal striatum (dSTR), nucleus accumbens (NAc), primary motor cortex (MC), amygdala (AMY) and ventral tegmental area (VTA) were isolated and dissected to analyze protein changes in additional brain areas with relevance to adolescent behavior. The coordinates for each region (relative to Bregma) were: +1.0–1.5 mm (± 0.2) for dSTR, NAc and M1, -0.9–1.4 mm (± 0.2) for AMY and -3.3–3.8 mm (± 0.2) for VTA.

Following dissection of regions of interest, tissue punches were homogenized by pulse sonication (4 s) in 10 mM Tris (pH 7.4 at 23°C) with 1% w/v SDS and 1:100 Halt EDTA-free Protease and Phosphatase Inhibitor Cocktail (Pierce, ThermoFisher Scientific, Rockford, IL). Brain tissue was stored at -80°C. The Pierce BCA kit (ThermoFisher Scientific) was used to determine protein concentrations (μg/ μL) of each tissue sample.

#### Immunoblots

Protein samples from each brain region, at 5 μg per sample, were run on a TGX 4–15% 18-well gel (BioRad) with 1x tris-glycine-SDS running buffer (Tris 25 mM, Glycine 192 mM, 0.1%SDS) with Protein Plus Dual Color (Bio-Rad) and See Blue ladders (ThermoFisher Scientific) and dry-transferred onto a PDVF membrane using the Invitrogen iBlot protocol (ThermoFisher Scientific). Membranes were blocked for 2 hours at room temperature in 3% bovine serum albumin (BSA, for STXBP1 and DPYSL2; Sigma-Aldrich), 1% BSA (actin) or 5% w/v non-fat dry milk (for DNM1 and CFL1; ThermoFisher Scientific). Membranes were incubated with the following primary antibodies overnight at 4°C with gentle rocking: rabbit polyclonal anti-STXBP1-1 [1:1000 in 3% BSA; Cell Signaling Technology, Inc., Danvers, MA], rabbit polyclonal anti-CFL1 [1:5000 in 5% non-fat dry milk, Cell Signaling Technology, Inc. [27]], mouse monoclonal anti-DNM1 [1:1000 in 5% non-fat dry milk, Cell Signaling Technology, Inc.]. Blots were incubated with rabbit polyclonal anti-DPYSL2 [1:10,000 in 3% BSA; AbCam, Cambridge, MA [28]] and mouse monoclonal anti-actin [1:5000; Millipore] for 1 hour at room temperature. Membranes were then extensively washed and incubated with an HRP-labeled goat-anti rabbit or goat-anti mouse secondary antibody (1:20,000 in the same blocking buffer as the primary antibody; Jackson ImmunoResearch Laboratories, Inc., West Grove, PA). Protein expression was assessed via an enhanced chemiluminescence protocol (Pierce ECL, ThermoFisher Scientific), with exposure to autoradiography film (Bio Express, Kaysville, UT). Protein bands were quantified by optical density analysis (NIH/Scion Image) and normalized to actin which was used as a loading control.

Antibody selectivity for the target protein was established by the vendor (example blots are available on the manufacturers’ websites.) Prior to immunoblot analysis of experimental tissue, blots with additional adolescent and adult mouse brain homogenate were probed with each antibody to validate the vendor’s findings. All antibodies chosen for the confirmation experiments showed a single band at the correct molecular weight marker for the indicated protein. Actin was chosen as a loading control for these experiments because it was not found to show differential expression between adolescent and adult mice in the mPFC in the proteomics screen. To confirm that this housekeeping protein was appropriate, adolescent and adult actin optical density was compared in all brain regions tested during analysis. No age differences in actin optical density emerged in any brain region.

#### Data analysis

Western blot data were analyzed using GraphPad Prism software (GraphPad Software, Inc, La Jolla, CA, v5). To determine the difference between adolescent and adult mice in the expression of proteins of interest, data were transformed to percent change in optical density from the adult control for each gel. As the immunoblotting represented a confirmation of the protemics data, significant differences were analyzed by a one-tailed unpaired t-test, with significance set *a priori* at *p* ≤ 0.05. Subjects with a percent change less than or greater than 2 standard deviations away from the group mean were considered outliers and were removed from the analysis (one adult mouse was removed from the STXBP1 and DNM1 blots, respectively).

## Results

### Proteomic analysis

To identify proteins with developmentally altered expression in the mPFC, brains from adolescent (PND 36) and adult (PND 80) mice were collected (Fig 1A). At the time of brain tissue collection, adolescent body weight was significantly lower than adult mice [*t*(22) = 8.10, *p* <0.0001] but brain weight was equivalent among the two age groups (*p* > 0.05; Fig 1B). The mPFC was dissected from each brain (Fig 1C), homogenized and labeled with red (adult) and green (adolescent) Cy dyes, run on 2D-DIGE, analyzed for expression differences between ages, and finally identified using tandem MALDI TOF/TOF mass spectrometry (Fig 1D).

The automated proteomic analysis spot picker detected 87 spots with differential expression in the adolescent and adult mPFC (Fig 1E). Manual curation to fulfil the criteria set (≥20% difference in all 4 gels) resulted in 58 differentially expressed spots, while an additional 4 spots were significantly altered at ≥15% across all 4 gels. All 62 spots were identified using MALDI-TOF and tandem TOF/TOF mass spectrometry (Table 1). mPFC data were analyzed as adolescent / adult expression, with positive fold change representing decreased protein expression in adolescents compared to adults and negative fold change representing increased protein expression in adolescents relative to adults. The majority of the identified proteins fell within the functional categories of cell-to-cell signaling, cell growth and motility, and cell metabolism.

**Table 1:** 62 Differentially expressed proteins in adult versus adolescent mPFC Identified in proteomics analysis.

Protein Name	Gene ID	Spot #	Peptide Count	Protein Score	Relative Change	*p* Value
Fatty acid-binding protein	FABP7	62	7	368	-2.14	< 0.0001
Neurocalcin-δ	NCALD	75	9	328	-1.76	< 0.0001
Dihydropyrimidinase-like 3	DPYSL3*	18	16	209	-1.72	< 0.0001
ATP synthase subunit delta	ATP5D	58	3	178	-1.70	< 0.0001
β-synuclein	SNCB	56	6	503	-1.68	0.0014
Dihydropyrimidinase-like 5	DPYSL5	23	22	675	-1.61	< 0.0001
Clathrin light chain A	CLTA	36	8	220	-1.60	< 0.0001
Cofilin-1	CFL1	54	9	158	-1.58	0.00028
Phosphoglycerate mutase 1	PGAM1	44	17	817	-1.54	0.0066
Membrane protein, palmitoylated 2 (MAGUK p55 subfamily member 2)	MPP2	17	19	421	-1.52	< 0.0001
Protein kinase C γ	PRKCG*	8	19	195	-1.51	0.013
Glyceraldehyde-3-phosphate dehydrogenase	GAPDH*	38	10	318	-1.51	0.0054
Protein phosphatase 3, regulatory subunit B, alpha	PPP3R1*	60	10	171	-1.48	0.0014
Growth associated protein 43	GAP43	31	9	201	-1.46	0.00048
Dihydropyrimidinase-like 4	DPYSL4	22	24	1030	-1.43	0.00022
Calreticulin	CALR	15	19	740	-1.42	0.00081
Dihydropyrimidinase-like 3	DPYSL3*	19	14	291	-1.42	< 0.0001
Protein phosphatase 3, regulatory subunit B, alpha	PPP3R1*	59	12	600	-1.40	0.011
Protein kinase C γ	PRKCG*	9	21	392	-1.40	0.021
Fascin actin-bundling protein 1	FSCN1	24	17	674	-1.37	< 0.0001
Drebrin	DBN1	72	23	858	-1.34	0.0018
Collapsin Response Mediator Protein 1	CRMP1	21	20	515	-1.33	< 0.0001
CB1 cannabinoid receptor-interacting protein 1	CNRP1	53	9	594	-1.32	0.033
3-oxoacid CoA transferase 1	OXCT1*	25	7	359	-1.32	< 0.0001
3-oxoacid CoA transferase 1	OXCT1*	26	14	891	-1.29	< 0.0001
Fatty acid-binding protein	FABP5	63	11	438	-1.28	< 0.0001
Voltage-dependent anion-selective channel protein 3	VDAC3	50	11	613	-1.27	0.0014
Dynamin-1	DNM1*	6	34	625	-1.25	0.0022
Synapsin II	SYN2	27	13	328	-1.24	0.0016
Guanine nucleotide binding protein (G protein), beta polypeptide 4	GNB4	37	15	231	-1.23	0.0028
HYDIN, axonemal central pair apparatus protein	HYDIN	82	21	41	-1.23	0.0078
Clathrin, light chain B	CLTB	74	13	424	-1.23	0.0096
Calbindin 2	CALB2	41	14	388	-1.22	0.014
3-hydroxybutyrate dehydrogenase, type 1	BDH1	49	12	489	-1.22	0.013
Fatty acid binding protein 3	FABP3	71	8	421	-1.20	0.0017
*Guanine nucleotide binding protein (G protein)*, *beta polypeptide 2-like 1*	*GNB2L1*	*68*	*18*	*972*	*-1*.*17*	0.00024
*Dihydropyrimidinase-like 2*	DPYSL2*	*14*	*28*	*878*	*1*.*14*	0.00088
*Enolase 2 (gamma*, *neuronal)*	*ENO2*	*30*	*19*	*861*	*1*.*18*	0.0019
*Creatine kinase*, *brain*	*CKB*	*32*	*21*	*881*	*1*.*18*	< 0.0001
Ubiquinol-cytochrome c reductase core protein II	UQCRC2	69	16	465	1.19	0.017
NADH dehydrogenase [ubiquinone] flavoprotein 1, mitochondrial	NDUFV1	77	18	380	1.20	< 0.0001
EF-hand domain-containing protein D2	EFHD2	40	10	302	1.21	0.004
Bridging integrator 1	BIN1	10	22	801	1.22	0.015
V-type proton ATPase subunit B, brain isoform	VATB2	81	12	87	1.23	0.0022
Pyruvate kinase isozymes M1/M2	KPYM	80	24	624	1.24	< 0.0001
Voltage-dependent anion-selective channel protein 1	VDAC1*	47	7	540	1.25	0.0015
Syntaxin-binding protein 1	STXB1*	79	26	716	1.26	0.0002
Guanine nucleotide-binding protein G(o) subunit α	GNAO1	35	13	647	1.27	< 0.0001
Glutathione S-transferase mu 5	GSTM5	73	18	639	1.28	< 0.0001
N-ethylmaleimide-sensitive factor	NSF	78	21	280	1.29	0.0078
Voltage-dependent anion-selective channel protein 1	VDAC1*	67	13	611	1.29	0.00029
Dynamin-1	DNM1*	4	22	338	1.31	0.00078
Septin-3	SEPT3	34	6	103	1.32	< 0.0001
Mitochondrial inner membrane protein	IMMT	7	28	625	1.32	0.00026
Carbonic anhydrase 2	CAH2	46	13	615	1.34	< 0.0001
Dihydropyrimidinase-like 2	DPYSL2*	13	25	719	1.35	0.00012
Cysteine and glycine-rich protein 1	CSRP1	51	8	334	1.36	0.017
Dihydropyrimidinase-like 2	DPYSL2*	12	26	800	1.38	< 0.0001
Syntaxin-binding protein 1	STXB1*	20	28	788	1.39	< 0.0001
Complexin-2	CPLX2	57	6	163	1.41	0.018
Septin-2	SEPT2	33	10	352	1.48	0.00058
Glyceraldehyde-3-phosphate dehydrogenase	GAPDH*	48	12	280	4.33	< 0.0001

58 spots showed ≤20% difference in expression in all 4 2D-DIGE gels (*p*<0.05; standard font), with an additional 4 spots with ≤15% difference in expression in all 4 2d-DIGE gels (*p*<0.05; italics). Each spot was identified via MALDI TOF/TOF mass spectrometry with a confidence of 1.0. Spot change was expressed as fold change ratio of adolescent from adult, with negative numbers reflecting an reduced expression in adults relative to adolescents and positive numbers indicating greater expression in adults. Asterisks denote spots that appear more than once in the proteomics report.

### Ingenuity pathway analyses

All proteins identified in the proteomics analysis were uploaded to Ingenuity Pathway Analysis for Global Functional Analysis (GFA) and Global Canonical Pathway (GCP) determination (Table 2). GFA revealed participation of identified proteins in cellular functions including cell-to-cell signaling and interaction, cellular morphology and cellular development. Additionally, identified proteins were shown to be involved in neurological disease, including schizophrenia and movement disorders. GCP analysis indicated the involvement of identified proteins in several known signaling cascades, including semaphoring signaling and axonal guidance signaling, androgen signaling, and glycolysis I and gluconeogenesis I signaling.

**Table 2:** Role of identified proteins in biofunctions and disorders, and canonical signaling networks.

**Biofunctions and disorders**			
**Function/Disorder**	***p*-Value**	**Higher in adults**	**Higher in adolescents**
***Cell-to-cell Signaling and Interaction***			
Synaptic transmission of synapse	0.000033	STXBP1,VDAC1	SNCB,VDAC3
Synaptic transmission of nervous tissue	0.0000549	NSF,STXBP1,VDAC1	SNCB,VDAC3
Synaptic transmission	0.00000124	NSF,STXBP1,VDAC1	PPP3R1,SNCB,SYN2,VDAC3
Long-term potentiation of synapse	0.00000198	CPLX2,VDAC1	CALB2,CRMP1,PPP3R1, PRKCG, VDAC3
Long-term potentiation	0.00000453	CPLX2,VDAC1	CALB2,CRMP1,DPYSL4, PPP3R1,PRKCG,VDAC3
Synaptic depression	0.0122		PPP3R1,PRKCG,SYN2
***Cellular Morphology***	***p*-Value**	**Higher in adults**	**Higher in adolescents**
Size of neurons	0.00333		DPYSL3,GAP43,SNCB
Morphology of neurites	0.0187	CKB	DPYSL4,GAP43
Morphology of neurons	0.00716	CKB,DNM1	CRMP1,DPYSL4,DPYSL5, GAP43
Length of neurites	0.00173	DPYSL2	DPYSL3,DPYSL4
Length of neurons	0.000214	DPYSL2	DBN1,DPYSL3,DPYSL4
Formation of filopodia	0.0000124	CSRP1	DPYSL3,DPYSL5
Extension of plasma membrane projections	0.0213	DPYSL2	DBN1,DPYSL5
Outgrowth of neurites	0.011	DPYSL2,GNAO1	DPYSL3,DPYSL5,GAP43
Branching of neurons	0.000225	CSRP1,DPYSL2	CRMP1,DBN1,DPYSL3,DPYSL4, GAP43
Morphogenesis of neurites	0.00276	CSRP1,DPYSL2,SEPT2	CRMP1,DBN1,DPYSL4,GAP43
Dendritic growth/branching	0.00244	CSRP1	CRMP1,DBN1,DPYSL4,GAP43
Branching of neurites	0.00125	CSRP1,DPYSL2	CRMP1,DBN1,DPYSL4,GAP43
Morphology of cells	0.00137	CKB,DNM1	CRMP1,DPYSL3,DPYSL4, DPYSL5,GAP43,SNCB
Size of brain	0.0248	CKB	FSCN1,GAP43
***Cellular Development***	***p*-Value**	**Higher in adults**	**Higher in adolescents**
Abnormal morphology of cerebral cortex	0.00591	CKB,CPLX2	CRMP1,GAP43
Abnormal morphology of brain	0.0138	CKB,CPLX2	CRMP1,GAP43,SNCB,SYN2
Abnormal morphology of nervous system	0.0332	CKB,CPLX2,DNM1	CRMP1,GAP43,SNCB,SYN2
Morphology of nervous system	0.00607	CKB,CPLX2,DNM1	CRMP1,DPYSL4,DPYSL5, GAP43,SNCB,SYN2
Proliferation of cells	0.00297	CSRP1,DPYSL2,GNAO1, PKM	CFL1,DPYSL3,DPYSL5,FABP7,GAP43
Proliferation of neuronal cells	0.00476	CSRP1,DPYSL2,GNAO1	CFL1,DPYSL3,DPYSL5,GAP43
Differentiation of cells	0.0014	CSRP1,DPYSL2	CRMP1,DBN1,DPYSL3,DPYSL4, FABP7,GAP43
Transport of synaptic vesicles	0.00000475	BIN1,CPLX2,DNM1,DPYSL2	SNCB
Endocytosis of synaptic vesicles	0.000186	BIN1,DNM1	SNCB
Growth of neurites	0.0056	CSRP1,DPYSL2,GNAO1	DPYSL3,DPYSL5,GAP43
***Neurological Disease***	***p*-Value**	**Higher in adults**	**Higher in adolescents**
Neuromuscular disease	0.000121	CA2,CKB,ENO2	DPYSL3,FABP7,GAP43, PPP3R1,PRKCG
Schizophrenia	0.00000118	ATP6V1B2,CSRP1,VDAC1	CLTB,GAP43,OXCT1,PGAM1, SNCB
Disorder of basal ganglia	0.000117	CA2,CKB,ENO2	DPYSL3,FABP7,GAP43, PPP3R1,PRKCG
Movement Disorders	0.00014	CA2,CKB,ENO2	DPYSL3,FABP7,GAP43, PPP3R1,PRKCG
Huntington's Disease	0.000119	CA2,CKB,ENO2	DPYSL3,FABP7,PPP3R1, PRKCG
**Canonical pathways and upstream regulators**			
**Pathways**	***p* Value**	**Higher in adults**	**Higher in adolescents**
Semaphorin Signaling in Neurons	2.31E-08	DPYSL2	CRMP1,CFL1,DPYSL3,DPYSL4, DPYSL5
Glycolysis I	7.01E-07	PKM,ENO2,GAPDH	PGAM1
Huntington's Disease Signaling	8.93E-07	DNM1,NSF,CPLX2	CLTA,CLTB,GNB2L1,GNB4, PRKCG
Androgen Signaling	4.05E-05	GNAO1	CALR,GNB4,GNB2L1,PRKCG
Gluconeogenesis I	4.84E-05	ENO2,GAPDH	PGAM1
Axonal Guidance Signaling	7.26E-05	DPYSL2,GNAO1	GNB4,CFL1,PPP3R1,GNB2L1, DPYSL5,PRKCG
**Regulators**			
MAPT	1.15E-16	CKB,CPLX2,DPYSL2,ENO2,GAPDH,GNAO1,PKM, STXBP1,VDAC1,	ATP5D,CFL1,CLTA,CLTB, PGAM1,SNCB
APP	3.88E-15	CKB,CPLX2,DNM1,DPYSL2,ENO2,GAPDH,GNAO1,PKM,STXBP1,VDAC1	ATP5D,CFL1,CLTA,CLTB,DBN1, FABP3,GAP43,PGAM1,SNCB, SYN2
PSEN1	7.74E-14	CKB,CPLX2,DPYSL2,ENO2,GAPDH,GNAO1,PKM, STXBP1,VDAC1	ATP5D,CFL1,CLTA,CLTB, PGAM1,SNCB
MKNK1	2.84E-08	CPLX2,GNAO1,STXBP1	CRMP1,DPYSL3,GAP43,SYN2
BDNF	6.22E-08	CPLX2,GNAO1,STXBP1	CALB2,CRMP1,DPYSL3,FSCN1,GAP43,SYN2

Proteomics results were analyzed via Ingenuity Pathway Analysis for known interactions with other proteins, signaling systems and networks in the Ingenuity Knowledge Base. Proteins that displayed higher expression during adulthood are shown on the left, and proteins that displayed higher expression during adolescence are shown on the right, as seen in Table 1. *p* values were derived from Ingenuity Pathway Analysis by right-tailed Fisher exact test and indicate relative overrepresentation of proteins in a given function compared with what is expected by chance.

### Network analysis and Western blot confirmation

Ingenuity Pathway Analysis was performed to identify functional protein networks likely to be impacted by the developmentally regulated proteins identified in the proteomics analysis. Three significant networks were identified: Network 1, cellular assembly and organization, cellular function and maintenance, and cellular movement (*p*-score 29); Network 2, behavior, cell signaling and interaction, nervous system development and function (*p*-score 31); Network 3, neurological disease, skeletal and muscular disorders, and psychological disorders (*p*-score 22). Complete Western blot gel images for all proteins tested are shown in supporting information figures (S1–S24 Figs).

#### Network 1

**Fig 2:**
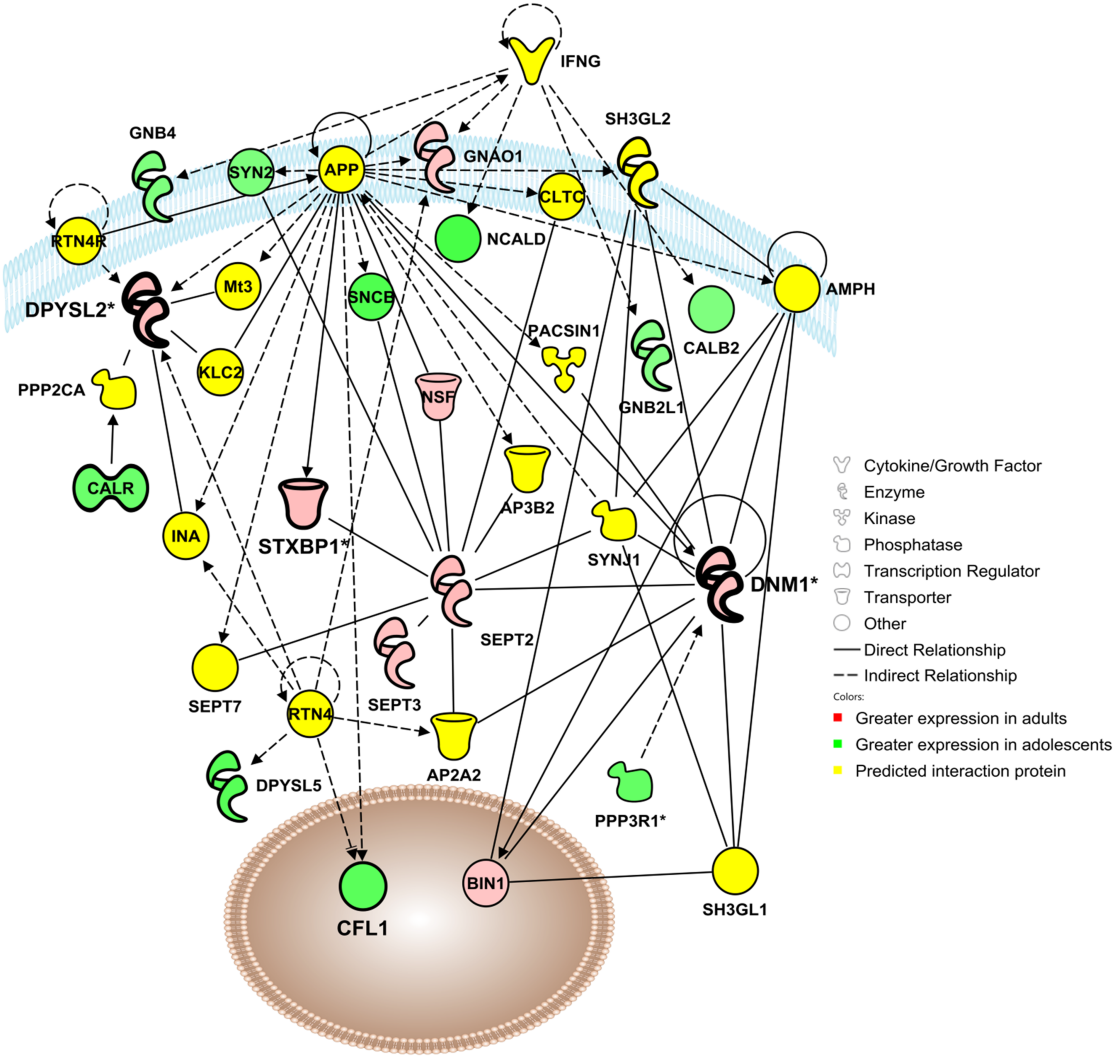
Adolescent development impacts a functional protein network involved in cellular assembly and organization, cellular function and maintenance, and cellular movement. Visualization of a protein interaction network identified by Ingenuity Pathway Analysis as being altered by adolescent brain development (*p*-score = 29). Proteins shown in red were up-regulated in the adult mPFC, proteins in green were up-regulated in the adolescent mPFC, and proteins in yellow indicate statistically significant interaction proteins identified by IPA network analysis. Solid lines indicate a direct interaction, and dashed lines indicate an indirect interaction mediated by additional, non-significant proteins. Asterisks denote proteins that were identified multiple times in the proteomic analysis that have been consolidated into a single point in the functional network. Molecules for confirmation are indicated in bold, e.g. syntaxin binding protein 1 (STXBP1), dynamin-1 (DNM1) and dihydropyrimidinase-like-2 (DPYSL2).

Network 1 (Cellular Assembly & Organization) includes 18 focus molecules identified in the proteomics analysis of adolescent and adult mPFC as well as 17 significant interaction proteins (Fig 2). Noteworthy predicted regulators of this network include interferon gamma (INFG) and reticulon 4 (RTN4).

**Fig 3:**
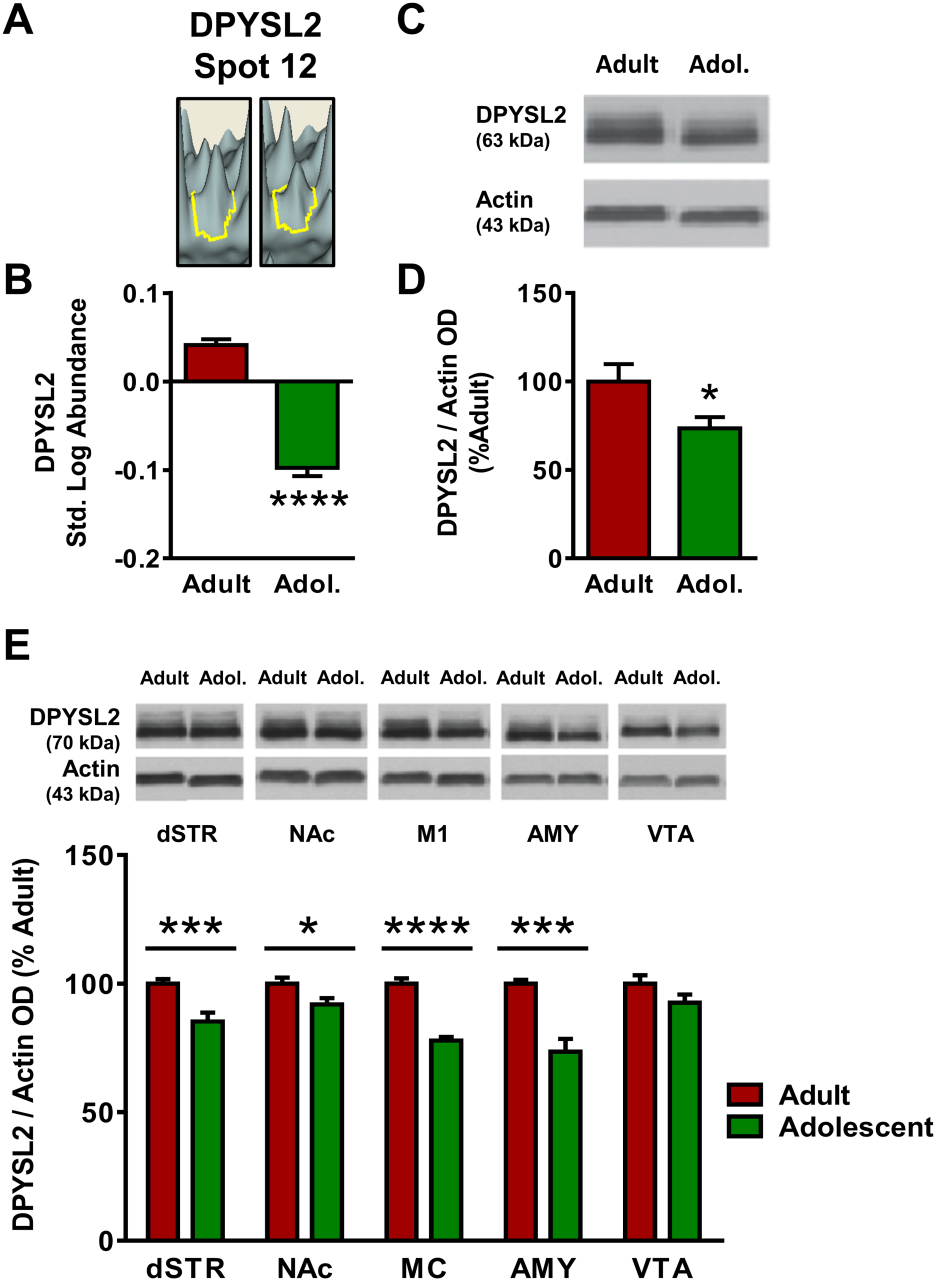
Adult and adolescent expression of dihydropyrimidinase-like-2 (DPYSL2). **(A)** Representative 3D plot of DPYSL2 expression in adult (left) and adolescent (right) mice for Spot #20. **(B)** Standardized abundance (log) of DPYSL2 demonstrating higher expression in adults versus adolescents. **(C)** Representative gel image of a Western blot for DPYSL2 expression to confirm 2D-DIGE changes. Both resulting bands were quantified. **(D)** Quantification of Western blot results, confirming reduced expression of DPYSL2 (normalized to actin) in adolescents as compared to adults. **(E)** Top, representative gel images for each brain region; bottom, quantification of Western blots for each brain region. Adults show increased expression of DPYSL2 in dStr, NAc, MC and Amy. No significant age differences were observed in the VTA (*p*>0.05). Data were expressed as percent change from mean adult within the same blot and graphed as mean ± SEM. (* indicates *p*≤0.05, *** indicates *p*≤0.001).

Of the focus molecules, dihydropyrimidinase-like 2 (DPYSL2) was a locus of significant interconnectedness in Network 1 and was thus selected for further analysis. DPYSL2 appeared at Spot 12 in the proteomics analysis (Fig 1E), where its standardized log abundance was 0.041 in adults and -0.098 in adolescents, representing a 38% reduction in adolescents versus adults (Fig 3A–3C). Spot 12 was identified as DPYSL2 with a protein score of 800 (Table 1), and no other candidate proteins met the pre-hoc criteria for identification at Spot 12. Western blot analysis confirmed the decrease observed in the proteomics analysis, with adolescent expression of DPYSL2 27% in mPFC lower than adult [*t*(26) = 2.27, *p* <0.05; Fig 3D and 3E].

In addition to the results of the proteomic analysis and Western blots in the mPFC, other brain regions were of interest based on their association with adolescent-typical behaviors. The dorsal striatum (dSTR), nucleus accumbens (NAc), primary motor cortex (MC), amygdala (AMY) and ventral tegmental area (VTA) were also analyzed for expression of the selected confirmation proteins. DPYSL2 expression was significantly reduced in adolescent mice as compared with adult mice in all brain regions examined excepting the VTA (*p* >0.05; Fig 3F). Age differences were most pronounced in the MC [22% decrease; *t*(23) = 9.00, *p* <0.0001] and AMY [26% decrease; *t*(19) = 4.45, *p* <0.0001], with less pronounced differences in the dSTR [18% decrease; *t*(22) = 3.59, *p* <0.001] and NAc [9% decrease; *t*(23) = 2.42, *p* <0.05].

**Fig 4:**
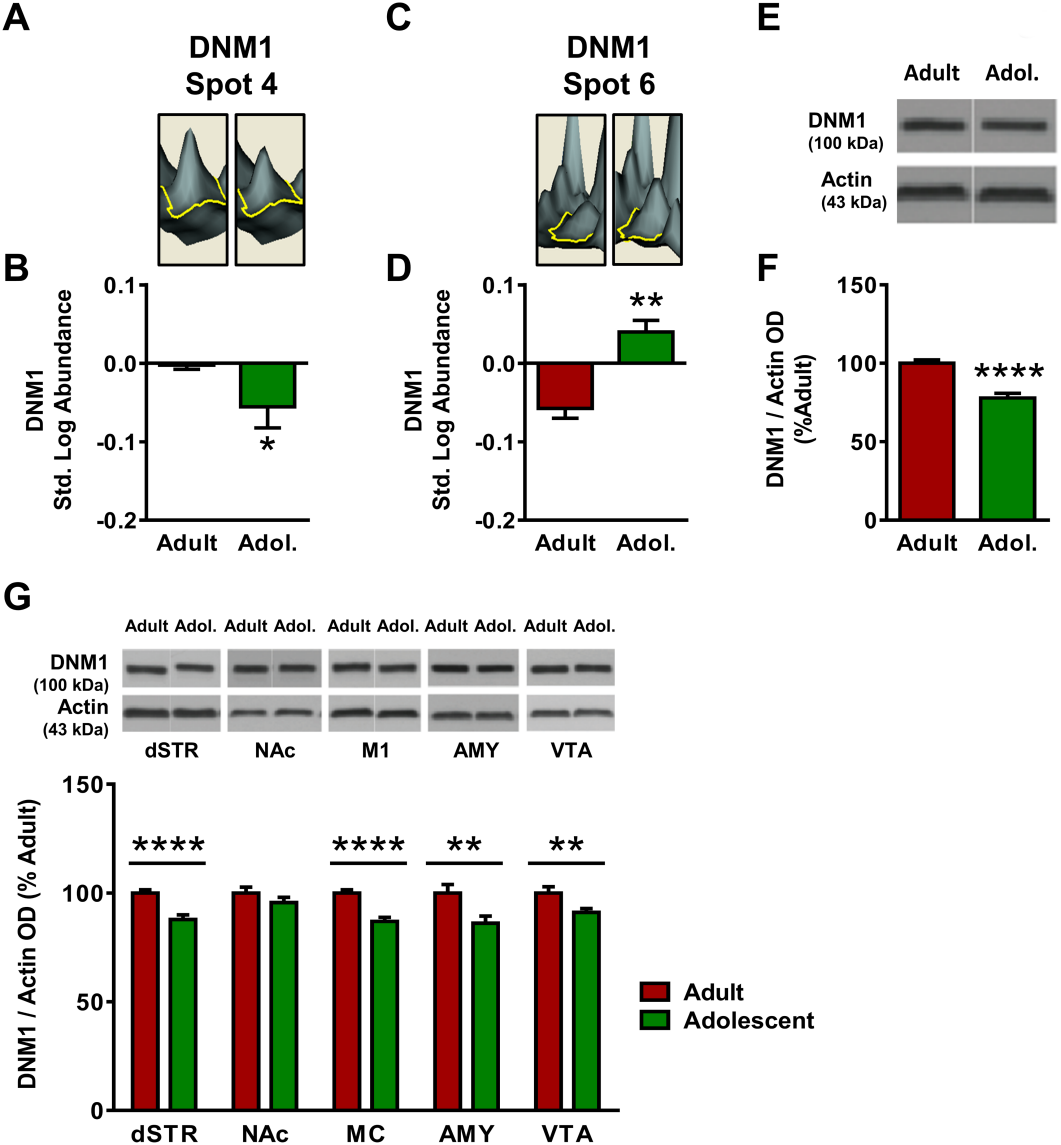
Adult and adolescent expression of dynamin-1 (DNM1). **(A)** Representative 3D plot of DNM1 expression in adult (left) and adolescent (right) mice for Spot #4. **(B)** Standardized abundance (log) of DNM1 (Spot 4) demonstrating higher expression in adults versus adolescents **(C)** Representative 3D plot of DNM1 expression in adult (left) and adolescent (right) mice for Spot #6. **(D)** Standardized abundance (log) of DNM1 (Spot 6) demonstrating higher expression in adults versus adolescents. **(E)** Representative gel image of a Western blot for DNM1 expression to confirm 2D-DIGE changes. **(F)** Quantification of Western blot results, confirming reduced expression of DNM1 (normalized to actin) in adolescents as compared to adults. **(G)** Top, representative gel images for each brain region; bottom, quantification of Western blots for each brain region. DNM1 expression was higher in adults in dStr, M1, Amy, and VTA. There was no significant change in DNM1 expression in NAc (p>0.05). Data were expressed as percent change from mean adult within the same blot and graphed as mean ± SEM. (* indicates *p*≤0.05, ** indicates *p*≤0.01, *** indicates *p*≤0.001, **** indicates *p*≤0.0001).

Dynamin-1 (DNM1) was also significantly interconnected in Network 1 and was subsequently analyzed. In the proteomics analysis DNM1 was identified twice, at Spot #4 and Spot #6 (Fig 1E). At Spot 4, DNM1 standardized log abundance was -0.003 for adults and -0.056 for adolescents, representing a 31% decrease in adolescent mice compared to adults (Fig 4A–4C). Spot 4 was identified as DNM1 with a peptide score of 338 (Table 1), and no other candidate proteins met the pre-hoc criteria for identification at Spot 12. At Spot 6, DNM1 standardized log abundance was -0.058 for adults and 0.400 for adolescents, representing a 26% increase in adolescent mice compared to adults (Fig 4D–4F). At Spot 6, the candidate proteins DNM1 and DNM2 both exceeded the pre-hoc criteria of 95% C.I., and DNM1 was selected as the identity for Spot 6 based on the higher peptide score for DNM1 (625) versus DNM2 (83). Western blot analysis indicated that DNM1 expression was decreased by 22% in adolescent mPFC as compared to adults [*t*(25) = 5.84, *p* <0.0001; Fig 4G and 4H]. DNM1 expression was also significantly lower in adolescents compared with adults in dSTR [12% decrease; *t*(22) = 4.48; *p* <0.0001], MC [13% decrease; *t*(23) = 5.48, *p* <0.0001], AMY (14% decrease; *t*(18) = 2.73, *p* <0.01] and VTA (9% decrease; *t*(21) = 2.68, *p* <0.01, Fig 4I]. The NAc displayed no age differences in DNM1 expression (*p* >0.05).

#### Network 2

**Fig 5:**
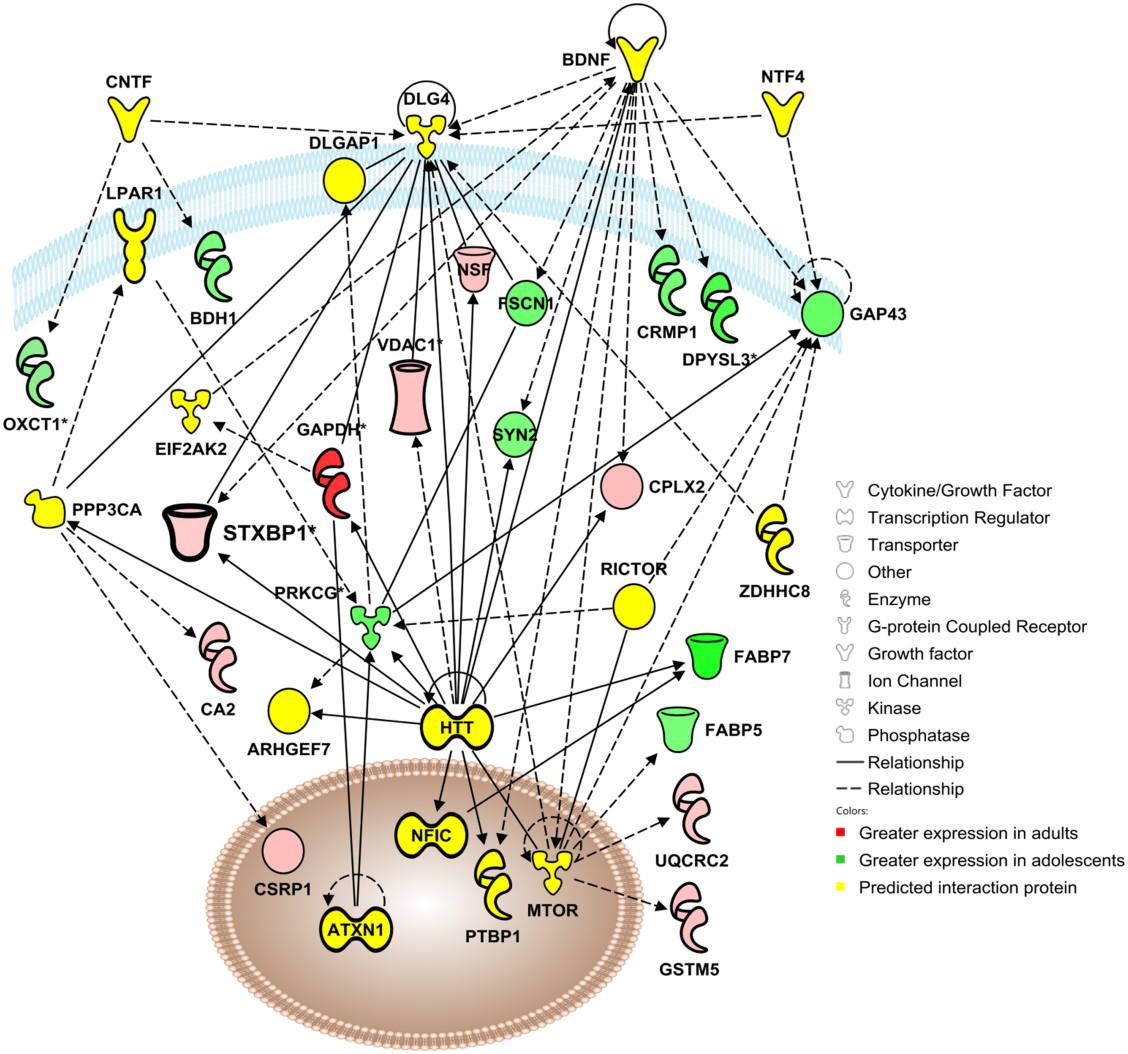
Adolescent development impacts a functional protein network involved in behavior, cell-to-cell signaling and interaction, and nervous system development and function. Visualization of a protein interaction network identified by Ingenuity Pathway Analysis as being altered by adolescent brain development (*p* score = 31). Proteins shown in red were up-regulated in the adult mPFC, proteins in green were up-regulated in the adolescent mPFC, and proteins in yellow indicate statistically significant interaction proteins identified by IPA network analysis. Solid lines indicate a direct interaction, and dashed lines indicate an indirect interaction mediated by additional, non-significant proteins. Asterisks denote proteins that were identified multiple times in the proteomic analysis that have been consolidated into a single point in the functional network. Focus molecule syntaxin binding protein 1 (STXBP1) is indicated in bold.

Network 2 (Behavior/Signaling) includes 19 focus molecules identified in the proteomics analysis as well as 16 proteins statistically predicted to interact with the focus proteins in a functional network (Fig 5). Of the focus molecules, 9 exhibited higher expression in adult mPFC whereas 10 exhibited greater expression in adolescents Notable predicted regulators of this network include brain-derived neurotrophic factor (BDNF) and huntingtin (HTT).

**Fig 6:**
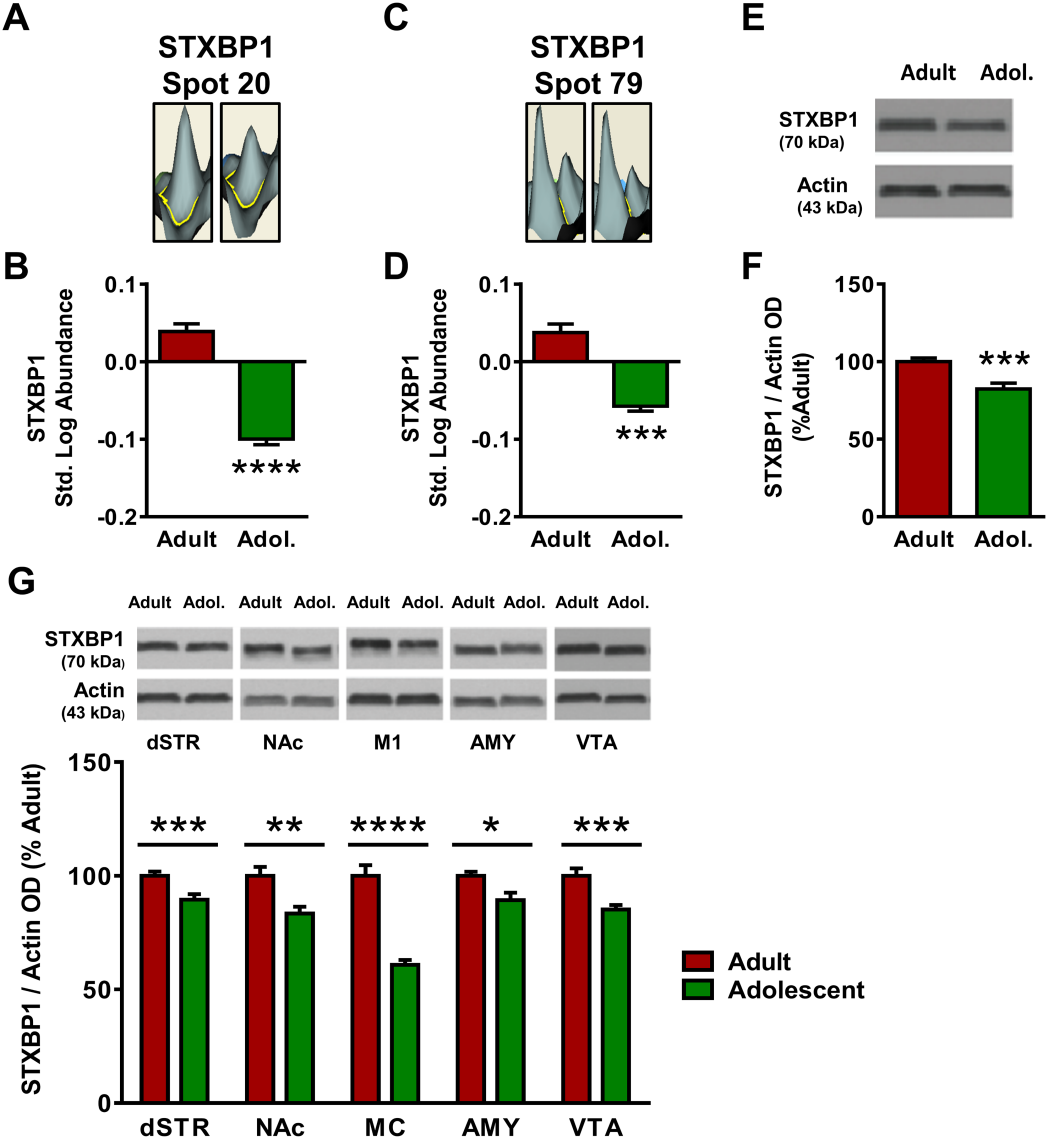
Adult and adolescent expression of syntaxin binding protein 1 (STXBP1). **(A)** Representative 3D plot of STXBP expression in adult (left) and adolescent (right) mice for Spot #20. **(B)** Standardized abundance (log) of STXBP1 (Spot 20) demonstrating higher expression in adults versus adolescents. **(C)** Representative 3D plot of STXBP1 expression in adult (left) and adolescent (right) mice for Spot #79. **(D)** Standardized abundance (log) of STXBP1 (Spot 79) demonstrating higher expression in adults versus adolescents. **(E)** Representative gel image of a Western blot for STXBP1 expression to confirm 2D-DIGE changes. **(F)** Quantification of Western blot results, confirming reduced expression of STXBP1 (normalized to actin) in adolescents as compared to adults. **(G)** STXBP1 expression was greater in adults in dStr, NAc, M1, Amy, and VTA. Top, representative gel images for each brain region; bottom, quantification of Western blots for each brain region. (*** indicates *p*≤0.001, **** indicates *p*≤0.0001.)

Of the focus molecules identified in the proteomics analysis in Network 2, syntaxin-binding protein 1 (STXBP1) was a significant hub of interconnectedness within the network and was therefore selected for further analysis. In the proteomics analysis of the mPFC STXBP1 was identified twice, at spot #20 and spot #79 (Fig 1E). At Spot 20, STXBP1 standardized log abundance was 0.039 for adults and -0.100 for adolescents, representing a 39% decrease in adolescent mice compared to adults (Fig 6A–6C). Spot 20 was identified as STXBP1 with a peptide score of 788, and no additional candidate proteins met the pre-hoc criteria for identification at that spot. At Spot 79, the candidate proteins STXBP1 and TPX2 both exceeded the pre-hoc criteria of 95% C.I., and STXBP1 was selected as the identity of Spot 79 based on the higher peptide score of STXBP1 (716) versus TPX2 (65). STXBP1 standardized log abundance was 0.036 for adults and -0.058 for adolescents, representing a 26% decrease in adolescent mice compared to adults (Fig 6D–6F). Western blot analysis confirmed the decreases observed in the proteomics analysis with adolescent expression of STXBP1 18% lower than adult expression in mPFC [*t*(25) = 3.89, *p* <0.001; Fig 6G and 6H]. STXBP1 expression was consistently reduced in adolescent mice as compared to adult mice in all brain regions examined (Fig 6I). The age difference was most pronounced in MC, where adolescent STXBP1 expression was decreased by 39% compared to adults [*t*(23) = 7.85, *p* <0.0001]. Age differences in the dSTR [11% decrease; *t*(23) = 3.98, *p* <0.001], NAc [17% decrease; *t*(23) = 3.35, *p* <0.01], AMY [11% decrease; *t*(18) = 2.48, *p* <0.05] and VTA [15% decrease; *t*(21) = 3.91, *p* <0.001] were more modest.

#### Network 3

**Fig 7:**
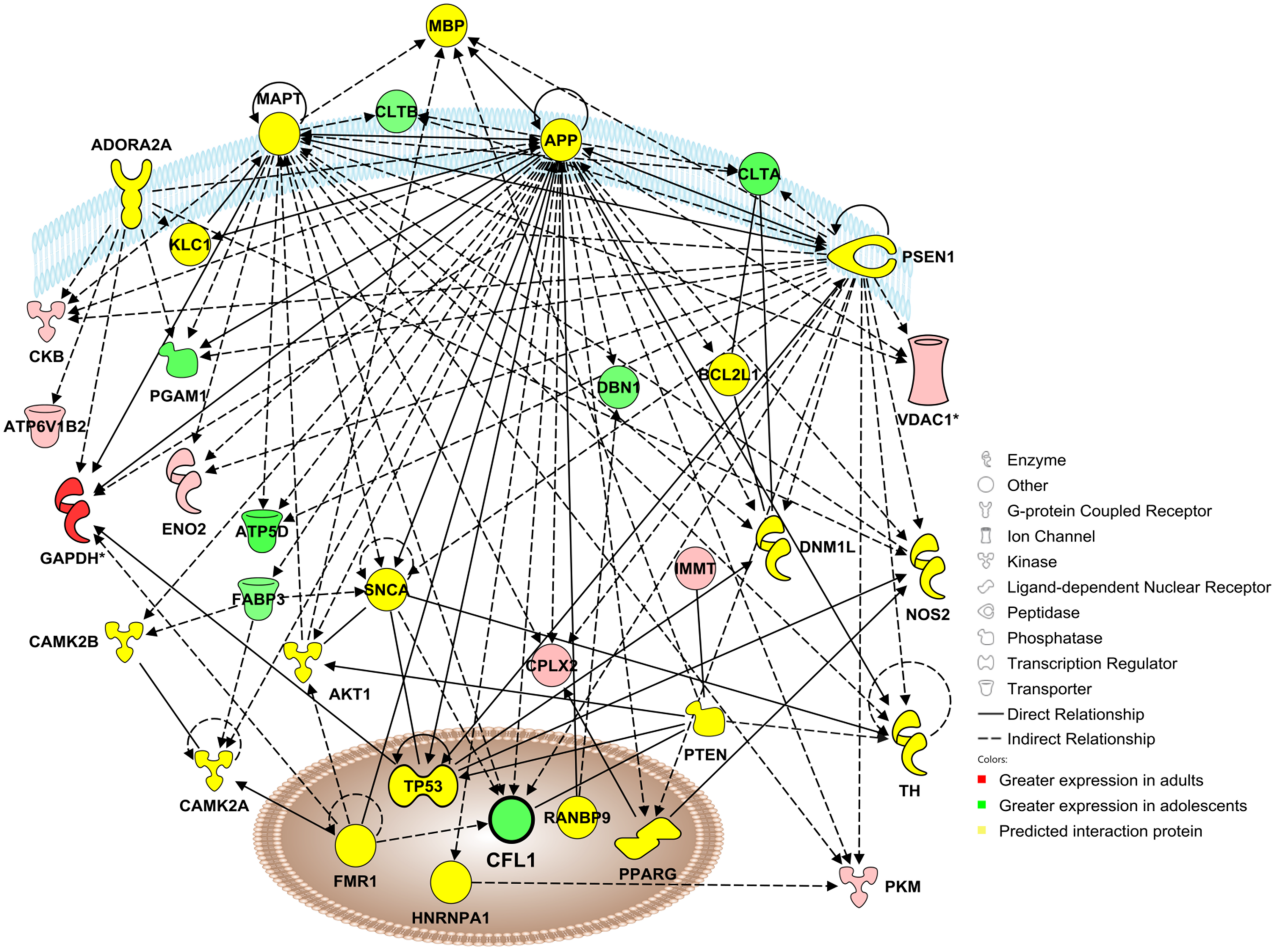
Adolescent development impacts a functional protein network involved with neurological disease, skeletal and muscular disorders, and psychological disorders. Visualization of a protein interaction network identified by Ingenuity Pathway Analysis as being altered by adolescent brain development (*p*-score = 22). Proteins shown in red were up-regulated in the adult mPFC, proteins in green were up-regulated in the adolescent mPFC, and proteins in yellow indicate statistically significant interaction proteins identified by IPA network analysis. Solid lines indicate a direct interaction, and dashed lines indicate an indirect interaction mediated by additional, non-significant proteins. Asterisks denote proteins that were identified multiple times in the proteomic analysis that have been consolidated into a single point in the functional network. Focus molecule cofilin-1 (CFL1) is indicated in bold.

Network 3 (Disease) includes 15 focus molecules identified in the proteomics analysis that were differentially expressed in adolescent as compared to adult mPFC as well as 20 significant interaction proteins (Fig 7). Of the focus molecules, 8 exhibited greater expression in adults whereas 7 had greater expression in adolescents. Significant predicted regulators of this network included amyloid-β precursor protein (APP), microtubule-associated protein tau (MAPT) and presenilin-1 (PSEN1).

**Fig 8:**
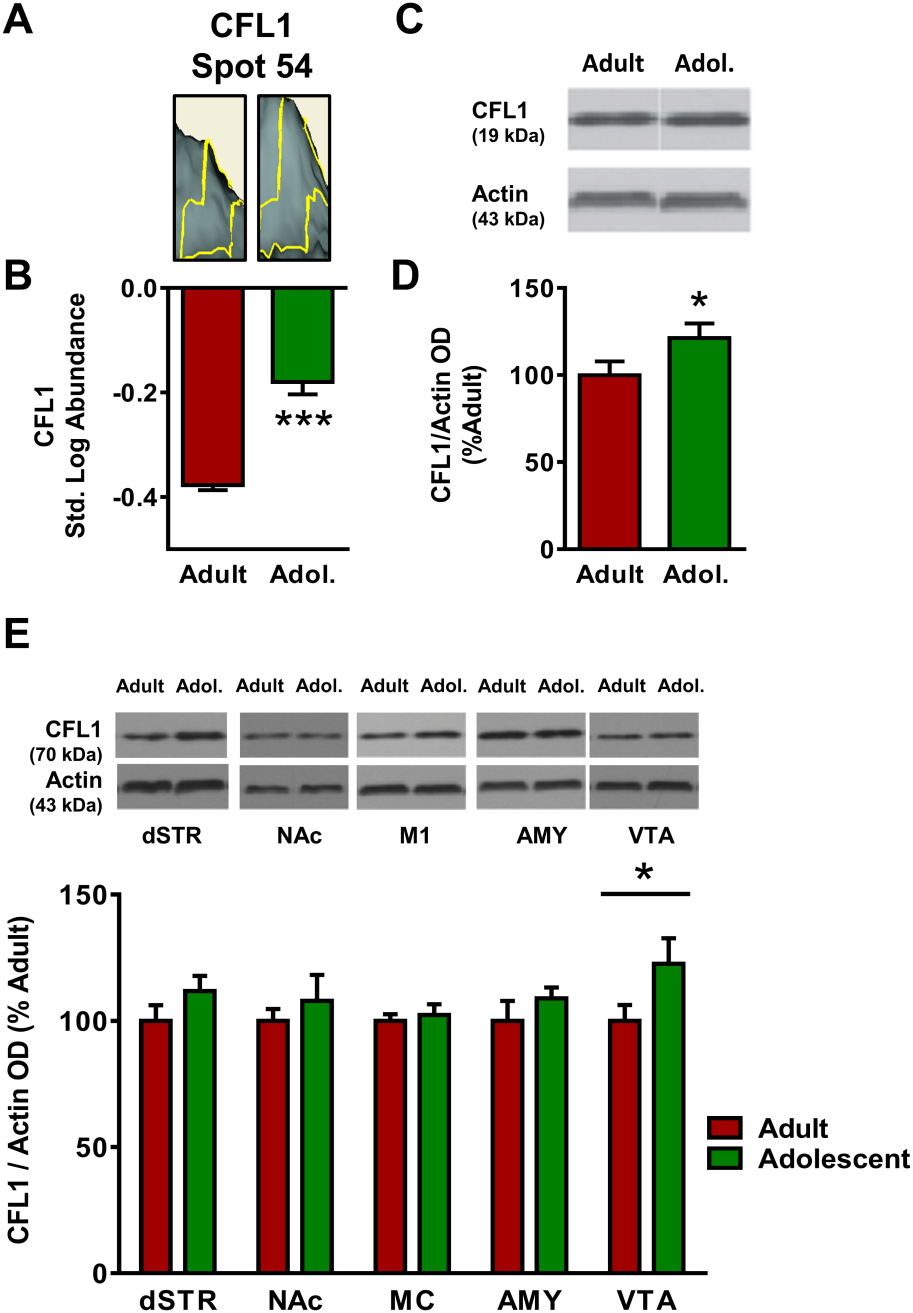
Adult and adolescent expression of cofilin-1 (CFL1). **(A)** Representative 3D plot of CFL1 expression in adult (left) and adolescent (right) mice for Spot #54. **(B)** Standardized abundance (log) of CFL1 demonstrating higher expression in adults versus adolescents. **(C)** Representative gel image of a Western blot for CFL1 expression to confirm 2D-DIGE changes. **(D)** Quantification of Western blot results, confirming reduced expression of CFL1 (normalized to actin) in adolescents as compared to adults. **(E)** Top, representative gel images for each brain region; bottom, quantification of Western blots for each brain region. Adolescents show higher expression of CFL1 in VTA. There were no significant changes in CFL1 expression in dSTR, NAc, MC or Amy (*p*>0.05). (* indicates *p*≤0.05, *** indicates *p*≤0.001).

The focus molecule cofilin-1 (CFL1) was a significant component of the network connectivity and was selected for further analysis. CFL1 appeared at Spot 54 in the proteomics analysis of the mPFC (Fig 1E), where its standardized log abundance was -0.378 in adults and -0.180 in adolescents, representing a 58% increase in adolescents versus adults (Fig 8A–8C). Both the candidate proteins CFL1 and CFL2 exceeded the pre-hoc criteria for identification, and CFL1 was chosen as the identity for Spot 54 based on the higher peptide score of CFL1 (158) versus CFL2 (149). Western blot analysis confirmed the increase observed in the proteomics analysis, with adolescent expression of CFL1 21% higher than adult [*t*(26) = 1.84, *p* <0.05; Fig 8D and 8E] in the mPFC. CFL1 expression was also significantly higher in adolescents versus adults in the VTA [23% increase; *t*(21) = 1.85, *p* <0.05; Fig 8F). No significant age differences in CFL1 emerged in the dSTR, NAc, MC or AMY (*p* >0.05).

## Discussion

Although significant structural changes in the prefrontal cortex during adolescence have been well established, the molecular changes that mediate these developmental alterations remain to be more fully characterized. The present study utilized a high-throughput unbiased proteomics approach to identify specific proteins and protein networks that show differential expression in adolescent compared to adult mPFC. 2D-DIGE followed by MALDI TOF/TOF identified 62 individual proteins with significant age-dependent differences in expression. Ingenuity Pathway Analysis identified 3 networks in which these target proteins were overexpressed. Further results confirmed key changes from previous investigations [20] while focusing the analysis on adolescent-specific protein changes, and extended these findings in additional brain regions (dSTR, MC, NAc, AMY and VTA). Together, the results indicate that, compared to adults, the adolescent mPFC has developmentally linked alterations in protein networks that regulate cellular organization/structure, neuronal signaling, anxiety-related behavior and neurological disease. These findings strengthen existing hypotheses about the progression of postnatal cortical development and point to several novel potential functional regulators of adolescent-typical behavior and vulnerability.

### Structural development

The present results provide several lines of evidence that suggest that the adolescent mPFC is characterized by widespread alterations in protein expression related to the regulation of cellular assembly & structure, cellular organization and structural plasticity. Many of the proteins identified in the proteomic analysis have roles in cellular morphology and synaptic plasticity. Ingenuity Global Canonical Pathway analysis also suggested that canonical signaling pathways known to regulate cellular growth and development in the adult mPFC were likely to differ from the adolescent condition, based on the interactions between proteins identified in the proteomics screen (Table 2). Both semaphorin signaling and axonal guidance signaling were identified as canonical signaling pathways that were impacted by the developmental state of the mPFC (Table 2). The majority of identified proteins in these canonical pathways were up-regulated in the adolescent cortex. Semaphorins are a family of receptors and secretory proteins that have a well-established role in guiding axonal outgrowth during embryonic development [29] and are also involved in neuronal maturation, synaptic plasticity and cell death in the adult cortex [30, 31]. Although the role of sempahorin signaling in adolescent brain maturation and synaptic pruning has not been investigated to date, this signaling system is a plausible mediator of morphological changes in adolescent cortex and merits examination in future studies.

Ingenuity Pathway Analysis identified a functional protein network involved in cellular assembly and organization that was significantly impacted by age (Fig 2). Several proteins identified in this network have been shown to play a role in synaptic development, including PP3R1 [32], CALR [33] and NSF [34], as well as the focus protein DPYSL2. DPYSL2 (or CRMP2) is a member of the CRMP family, which binds tubulin heterodimers to facilitate microtubule assembly [35]. These proteins function in growth cone formation, contributing to neuronal outgrowth [36] and may also play a role in cell death [37] and thus neural pruning. The expression of CRMP1 and DPYSL3, 4, and 5 was higher in the adolescent mPFC, consistent with increased neuronal outgrowth and synaptic formation during this developmental period (Table 1). However, DPYSL2 expression was found to be consistently higher in adults in the mPFC, dSTR, NAc, M1, and AMY (Fig 4). The reason why this CRMP subtype, but not the others, is up-regulated in adulthood is unknown, but could be due to a developmental shift from dominant expression of CRMP 1, DPYSL3, 4, or 5 in the adolescent brain to DPYSL2 in the adult brain. DPYSL2 was identified three times in the proteomic screen, at spots #12, 13 and 14. Spots 13 and 14 appeared at the same molecular weight range but exhibited different isoelectrical focusing, which may suggest a posttranslational modification affecting one of the two spots. Spot #12 exhibited shifts in both isoelectrical focusing and molecular weight, which could indicate a possibility of protein contamination, significant posttranslational modification, or both. Immunoblots confirmed the increase in total DPYSL2 in the adolescent mPFC, but additional assessment with antibodies targeting posttranslational modifications of DPYSL2 would provide clarity as to the variable spots detected in the proteomics screen.

### Signaling and behavior

In addition to their roles as mediators of cellular assembly and development, many of the proteins identified in the proteomics analysis are known to be involved in cell-to-cell signaling and neurotransmission. Indeed, Ingenuity Pathway Analysis identified a significant protein interaction network associated with cell signaling and behavior that was impacted by adolescent brain maturation (Fig 4). The immaturity of the prefrontal cortex has been suggested to underlie many adolescent-typical behaviors, such as impulsivity, reward sensitivity and risk taking. Subcortical areas involved in emotional processing (such as the nucleus accumbens, ventral tegmental area and amygdala) reach adulthood before regulatory control of these regions from the PFC is fully mature [16], leading to enhanced response to reward and impairments in inhibitory control under emotionally salient conditions [38].

At the cellular level, previous studies have demonstrated that the adolescent mPFC has altered responses to neurotransmitters and cell signaling molecules, including dopamine [39, 40], glutamate [41] and GABA [42]. Moreover, several studies have provided evidence for the regulation of adolescent-typical behaviors by diverse signaling systems including the cannabinoid systems [43], glutamate [44], dopamine [45] and GABA [46]. Within the network identified by IPA, several proteins with established roles in adolescent-related behaviors were observed, including BDNF [47, 48], GAP43 [49] and mTOR [50]. Regulation of both PFC function and behavior by these varied signaling systems may reflect large-scale structural changes occurring in the adolescent forebrain during development, consistent with the alterations in structural proteins we observed.

The focus proteins STXBP1 and DNM1 have both been shown to be involved in neurotransmitter signaling, and both displayed different expression patterns in the adolescent and adult mPFC. STXBP1 contributes to the regulation of exocytosis in cells, assisting in vesicle fusion and neurotransmitter release [51]. The proteomics screen revealed two individual spots that were each identified as STXBP1 (spot #20 and #79), both of which displayed decreased expression in adolescents versus adults (Fig 6). While it is possible that these multiple spots could represent contamination with other proteins, each spot was identified as STXBP1 with high confidence; spot #20 was identified as STXBP1 with 28 peptides and a peptide score of 788, and spot #79 was identified as STXBP1 with 26 peptides and a peptide score of 716. Based on these identifications, another explanation for the multiple identifications of STXBP1 in the proteomics screen is post-translational modifications. The presence of phosphate groups, methylation and other post-translational modifications can shift the isoelectrical focusing of a spot without altering the observed molecular weight or peptide identification. Immunoblotting confirmed that total STXBP1 expression is reduced in the adolescent mPFC relative to adults. This alteration in STXBP1 expression in the adolescent mPFC was also observed in each additional brain region examined (dStr, NAc, M1, Amy and VTA), which may indicate that this developmental change in STXBP1 levels is part of a brain-wide process, perhaps in response to the widespread changes in neurotransmitter signaling reported throughout the brain during adolescence [2]. The increased STXBP1 observed in the mature brain may contribute to greater regulation of synaptic transmission in adults, and ultimately greater refinement in the executive functioning of the PFC.

DNM1 participates in vesicle budding in both clathrin-mediated endocytosis and activity-dependent bulk endocytosis [52, 53], and mutations in dynamin cause developmental abnormalities in *drosophila* [54]. Proteomic analysis of DNM1 revealed two individual spots that were each identified as DNM1 (spot #4 and #6) As was the case for STXBP1, each spot was identified as DNM1 with high confidence; spot #4 was identified as DNM1 with 22 peptides and a peptide score of 338, and spot #6 was identified as DNM1 with 34 peptides and a peptide score of 625. Unlike STXBP1, however, in this instance one DNM1 spot displayed increased expression in the adult mPFC (spot #4) while the other showed increased expression in the adolescent mPFC (spot #6). To resolve this apparent contradiction, immunoblotting for total DNM1 protein showed that DNM1 expression was lower in the adolescent versus the adult mPFC, consistent with spot #4. The increased expression in DNM1 at spot #6 may indicate that a posttranslational modification of DNM1 has higher expression in the adolescent mPFC, perhaps in compensation for reduced protein levels. Future studies should examine the phosphorylation state and activity of DNM1 in the adolescent and adult cortex to test this hypothesis. Similar to STXBP1, Western blot analysis of total DNM1 expression in other brain regions showed higher DNM1 expression in adults across most brain regions, with the exception of the NAc where levels did not significantly differ between the two ages. Much like STXBP1, the generally elevated expression of DNM1 in the adult brain may represent more mature control of receptor expression at the cell surface and therefore refinement of synaptic signaling.

Whereas DPYSL2, STXBP1 and DNM1 showed a general decrease in the adolescent brain relative to the adult brain, the expression of the focus protein cofilin-1 (CFL1) was found to have higher expression in the adolescent prefrontal cortex in the proteomics analysis. Furthermore, the age-dependent alteration in CFL1 expression was relatively selective for the mPFC; of the additional brain regions tested, only the VTA showed a similar age difference (Fig 8). Spot 54 was identified as CFL1, but the 3D image rendered by DeCyder revealed a shoulder onto another spot, and the candidate protein CFL2 was also a significant identity for Spot 54. Ultimately CFL1 was chosen as the protein identity for Spot 54 based on the higher protein score of CFL1 versus CFL2, and immunoblotting experiments confirmed that the expression of CFL1 is increased in the adolescent mPFC versus the adult. However, the potential for CFL2 in addition to CFL1 being altered during adolescent brain development remains a strong possibility.

Both cofilin-1 and -2 are actin depolymerizing proteins [55] that contribute to spine growth and shrinkage [56, 57], cell migration [58] and AMPAR and NMDAR trafficking during both long-term potentiation (LTP) and long-term depression (LTD) [59, 60]. These observations make cofilin a particularly interesting protein in the context of adolescent development of the cortex and the developmental disorders associated with adolescent cortical disruption, such as schizophrenia.

Schizophrenia is a psychotic disorder characterized by significant impairments in cognition, hallucinations and delusions, and social withdrawal and mood disturbances [61] that is usually diagnosed during adolescence [62]. A significant neurobiological component of schizophrenia appears to be alterations in cortical gray matter; schizophrenia patients present with both faster reductions in cortical gray matter during adolescence [63] and greater total volume reductions in the prefrontal cortex [64], which may predate symptom onset [65]. Decreased dendritic spines [66] and synaptic markers [67] have also been reported in the schizophrenic cortex. Taken together, these findings suggest a “hyper-adolescent” state in the schizophrenic cortex, such that adolescent-typical neural pruning occurs in excess [68], eliminating necessary synaptic connections in the PFC and reducing ability to regulate brain function and behavior.

Several recent reports have linked actin dynamics and cofilin to schizophrenia [69, 70]. Additionally, Ingenuity Global Functional Analysis revealed significant enrichment of the identified protein dataset for proteins previously shown to be linked to schizophrenia, including GAP43 [71], VDAC1 [72] and SNCB [73], all of which have been shown to play a role in synaptic communication. These results underscore the association between normal adolescent brain development and pathology. However, neither the protein identifications nor the bioinformatics utilized in the present studies were able to assess a functional role for the observed protein alterations either in normal development or in disease. A major goal for future work will be the evaluation of protein targets like cofilin that may regulate adolescent brain development and be mechanistically involved in the etiology of developmental disorders such as schizophrenia.

### Limitations and future directions

The most significant limitation of the present work was the inability to assess the functional relevance of protein changes observed in these experiments. Although the identified proteins and subsequent bioinformatics analyses provide insight into the biochemical processes that may underlie the development occurring in the adolescent mPFC, these results are correlational and do not directly address the causative relationship, if any, between protein expression changes and structural/functional maturation. However, the high-throughput nature of the proteomics analysis enabled the identification of several novel targets and signaling pathways, providing significant heuristic value for subsequent investigations. The Ingenuity Pathway Analysis provided useful insight into additional protein targets whose activity, but not expression, may be altered during adolescent brain development, but the suggested impact of development on predicted interaction proteins was not directly assessed in these experiments. Future work should confirm changes in the activity or phosphorylation state of protein predicted by IPA to be altered in the adolescent mPFC.

The proteomic screen was conducted using 2D-DIGE and MALDI TOF/TOF MS, a high-throughput and cost-effective means of obtaining a “snapshot” of the mPFC proteome in adolescent and adult samples. However, gel-based proteomics applications have important limitations, the most significant of which are the inability to detect low-abundance proteins and the difficulty of resolving membrane-bound proteins [74]. Several previous studies have examined the synaptic fraction of the mPFC proteome in developing rodents [17–20], thus the present findings add to a preexisting literature by shedding light on the whole-cell protein alterations occurring during adolescent brain maturation. Still, the methods used in these experiments leave open the strong possibility that additional proteins not detected here may be altered during cortical development.

Additionally, the immunoblots were developed with radiography, which presents limited resolution compared with digital imaging systems. Subsequent experiments to expand the present findings could strengthen the conclusions reported herein by combining MS-based proteomics with digital imaging of immunoblots to confirm the protein expression changes observed. In the immunoblot experiments, actin was selected as a housekeeping protein due to its previous use as a loading control in development proteomics screens [18] and the lack of evidence for age differences in actin expression in the present proteomics assessment. Across all brain regions and proteins tested, the optical density of actin was not different between adolescents and adults. Nevertheless, the actin values were not compared to total protein in the immunoblot experiments, leaving open the possibility that loading errors could have occluded a genuine age difference, rending actin inappropriate as a housekeeping protein. However, four previous proteomics screen have failed to identify actin as developmentally altered during adolescent brain development [17–20]. Combined with the consistent lack of age differences in actin optical density observed in each brain region tested, the possibility of occult age differences in actin expression remains but seems unlikely.

During tissue collection, mice were perfused using 1.0M PBS to remove blood that could potentially contaminate brain tissue and alter the protein differences reported in the proteomics screen. However, this treatment entailed the use of an injected anesthetic, and injection stress could have altered the expression of identified proteins, as could the perfusion process itself. To avoid identifying age differences in proteins based on stress/perfusion alone, both adolescent and adult samples were treated identically during anesthesia and perfusion. Still, protein abundance in both ages could have been altered by these procedures.

### Conclusion

The present findings supplement an existing body of work that suggests that adolescence is characterized by enhancement of neural pruning, synaptic plasticity, and morphological changes as the brain matures into adulthood. Importantly, many of the proteins identified in the current experiments have previously been observed to be altered at the protein level in mouse cortex [20] and at the gene expression level in the postmortem human adolescent cortex [75]. The results of the proteomics analysis provide novel insight into a wider array of molecular alterations that may underlie the large-scale alterations in cortical gray matter and connectivity observed during adolescence. Further, functional protein networks involved in cellular assembly and signaling point toward pathways for future research into the mechanistic regulation of postnatal brain development. Future experiments to evaluate the role of the identified proteins and signaling systems in adolescent brain maturation, particularly in the context of psychiatric diseases such as schizophrenia, will shed further light on this crucial developmental period.

## Supporting information

S1 Fig DPYSL2 prefrontal cortex Western blots (DPYSL2-PFC). Photos represent the left and right sides of a single 18-lane membrane.
(TIF)

S2 Fig DPYSL2 dorsal striatum Western blots (DPYSL2-dSTR). Photos represent the left and right sides of a single 18-lane membrane.
(TIF)

S3 Fig DPYSL2 nucleus accumbens Western blots (DPYSL2-NAc). Photos represent the left and right sides of a single 18-lane membrane.
(TIF)

S4 Fig DPSYL2 motor cortex Western blots (DPYSL2-MC). Photos represent the left and right sides of a single 18-lane membrane.
(TIF)

S5 Fig DPYSL2 amygdala Western blots (DPYSL2-AMY). Photos represent the left and right sides of a single 18-lane membrane.
(TIF)

S6 Fig DPSYL2 ventral tegmental area Western blots (DPYSL2-VTA). Photos represent the left and right sides of a single 18-lane membrane.
(TIF)

S7 Fig STXBP1 prefrontal cortex Western blots (STXBP1-PFC). Photos represent the left and right sides of a single 18-lane membrane. Visible bands at ~100 kDa are Drebrin which was probed on the same blot.
(TIF)

S8 Fig STXBP1 dorsal striatum Western blots (STXBP1-dSTR). Photos represent the left and right sides of a single 18-lane membrane.
(TIF)

S9 Fig STXBP1 nucleus accumbens Western blots (STXBP1-NAc). Photos represent the left and right sides of a single 18-lane membrane.
(TIF)

S10 Fig STXBP1 motor cortex Western blots (STXBP1-MC). Photos represent the left and right sides of a single 18-lane membrane.
(TIF)

S11 Fig STXBP1 amygdala Western blots (STXBP1-AMY). Photos represent the left and right sides of a single 18-lane membrane.
(TIF)

S12 Fig STXBP1 ventral tegmental area Western blots (STXBP1-VTA). Photos represent the left and right sides of a single 18-lane membrane.
(TIF)

S13 Fig DNM1 prefrontal cortex Western blots (DNM1-PFC). Photos represent the left and right sides of a single 18-lane membrane. Visible bands at ~20 kDa are CFL1 which was probed on the same blot.
(TIF)

S14 Fig DNM1 dorsal striatum Western blots (DNM1-dSTR). Photos represent the left and right sides of a single 18-lane membrane. Visible bands at ~20 kDa are CFL1 which was probed on the same blot.
(TIF)

S15 Fig DNM1 nucleus accumbens Western blots (DNM1-NAc). Photos represent the left and right sides of a single 18-lane membrane. Visible bands at ~20 kDa are CFL1 which was probed on the same blot.
(TIF)

S16 Fig DNM1 motor cortex Western blots (DNM1-MC). Photos represent the left and right sides of a single 18-lane membrane. Visible bands at ~20 kDa are CFL1 which was probed on the same blot.
(TIF)

S17 Fig DNM1amygdala Western blots (DNM1-AMY). Photos represent the left and right sides of a single 18-lane membrane. Visible bands at ~20 kDa are CFL1 which was probed on the same blot.
(TIF)

S18 Fig DNM1 ventral tegmental area Western blots (DNM1-VTA). Photos represent the left and right sides of a single 18-lane membrane. Visible bands at ~20 kDa are CFL1 which was probed on the same blot.
(TIF)

S19 Fig CFL1 prefrontal cortex Western blots (CFL1-PFC). Photos represent the left and right sides of a single 18-lane membrane. Visible bands at ~100kDa are DNM1 which was probed on the same blot.
(TIF)

S20 Fig CFL1 dorsal striatum Western blots (CFL1-dSTR). Photos represent the left and right sides of a single 18-lane membrane. Visible bands at ~100kDa are DNM1 which was probed on the same blot.
(TIF)

S21 Fig CFL1 nucleus accumbens Western blots (CFL1-NAc). Photos represent the left and right sides of a single 18-lane membrane. Visible bands at ~100kDa are DNM1 which was probed on the same blot.
(TIF)

S22 Fig CFL1 motor cortex Western blots (CFL1-MC). Photos represent the left and right sides of a single 18-lane membrane. Visible bands at ~100kDa are DNM1 which was probed on the same blot.
(TIF)

S23 Fig CFL1 amygdala Western blots (CFL1-AMY). Photos represent the left and right sides of a single 18-lane membrane. Visible bands at ~100kDa are DNM1 which was probed on the same blot.
(TIF)

S24 Fig CFL1 ventral tegmental area Western blots (CFL1-VTA). Photos represent the left and right sides of a single 18-lane membrane. Visible bands at ~100kDa are DNM1 which was probed on the same blot.
(TIF)
