# Supplementary figures and images for: Comparison of the adolescent and adult mouse prefrontal cortex proteome
Source: PLoS One. 2017 Jun 1;12(6):e0178391. doi: 10.1371/journal.pone.0178391 (PMC5453624; doi:10.1371/journal.pone.0178391)

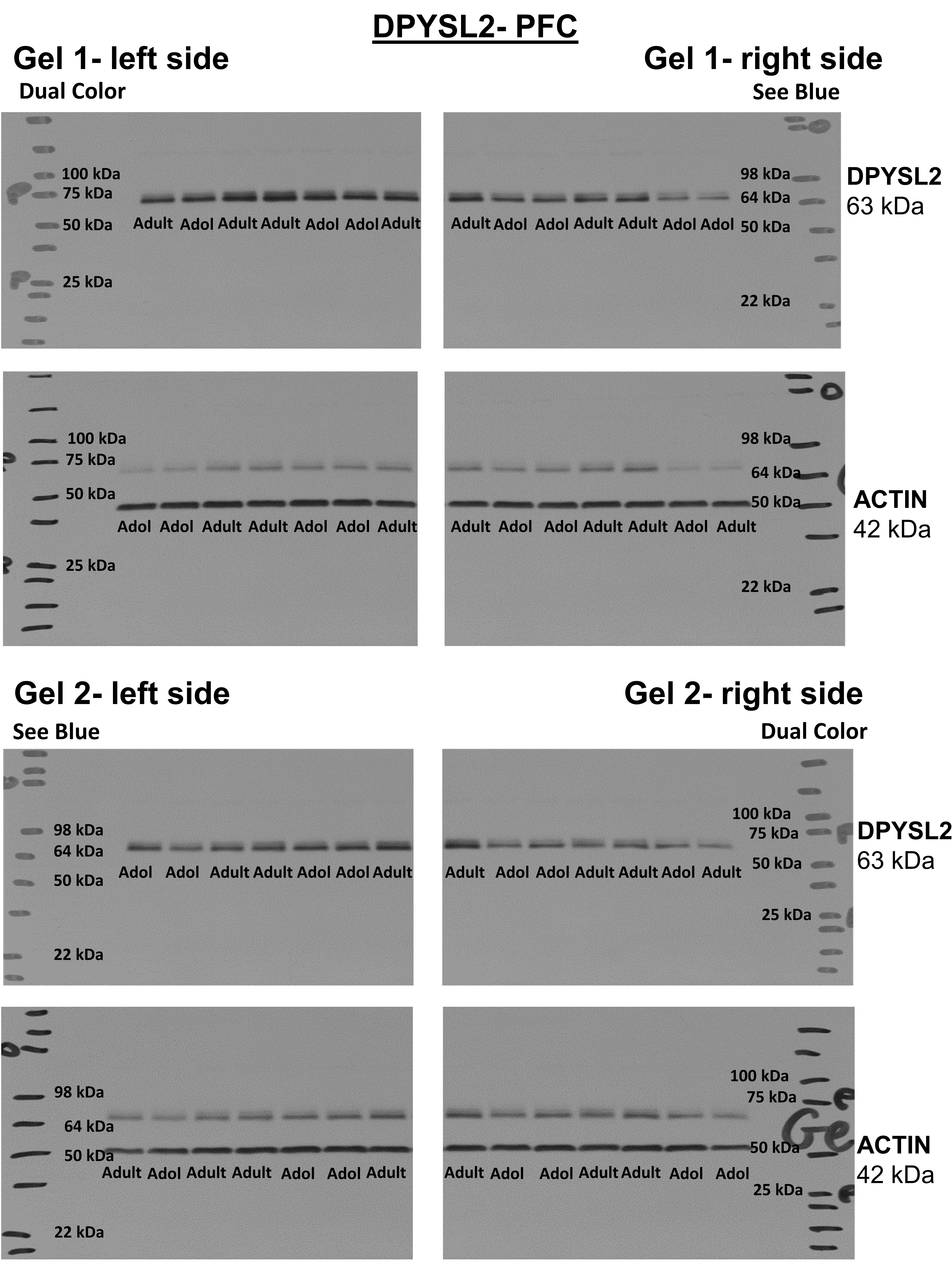

Supplement: S1 Fig — Photos represent the left and right sides of a single 18-lane membrane. (TIF) [file pone.0178391.s001.TIF]

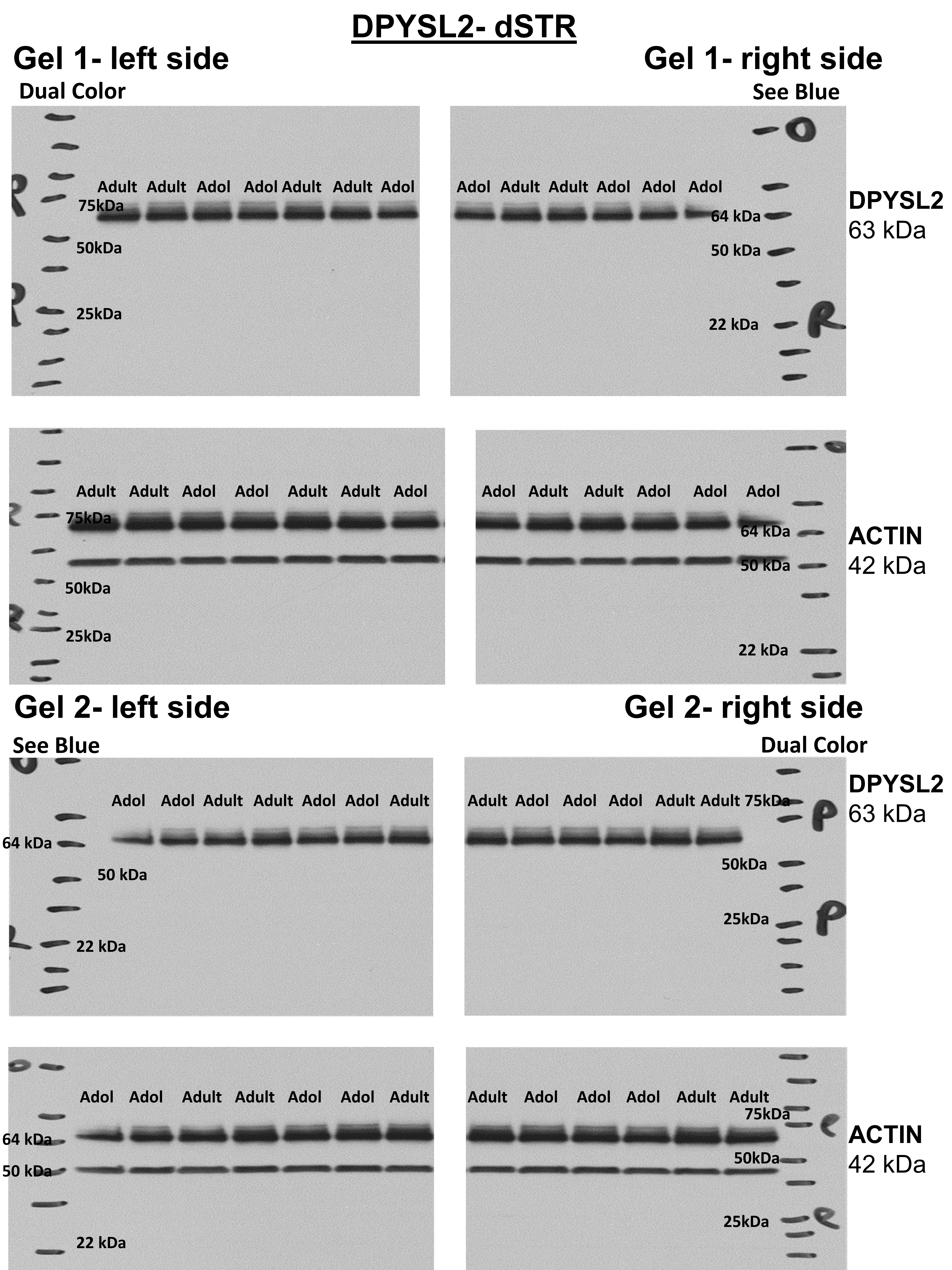

Supplement: S2 Fig — Photos represent the left and right sides of a single 18-lane membrane. (TIF) [file pone.0178391.s002.TIF]

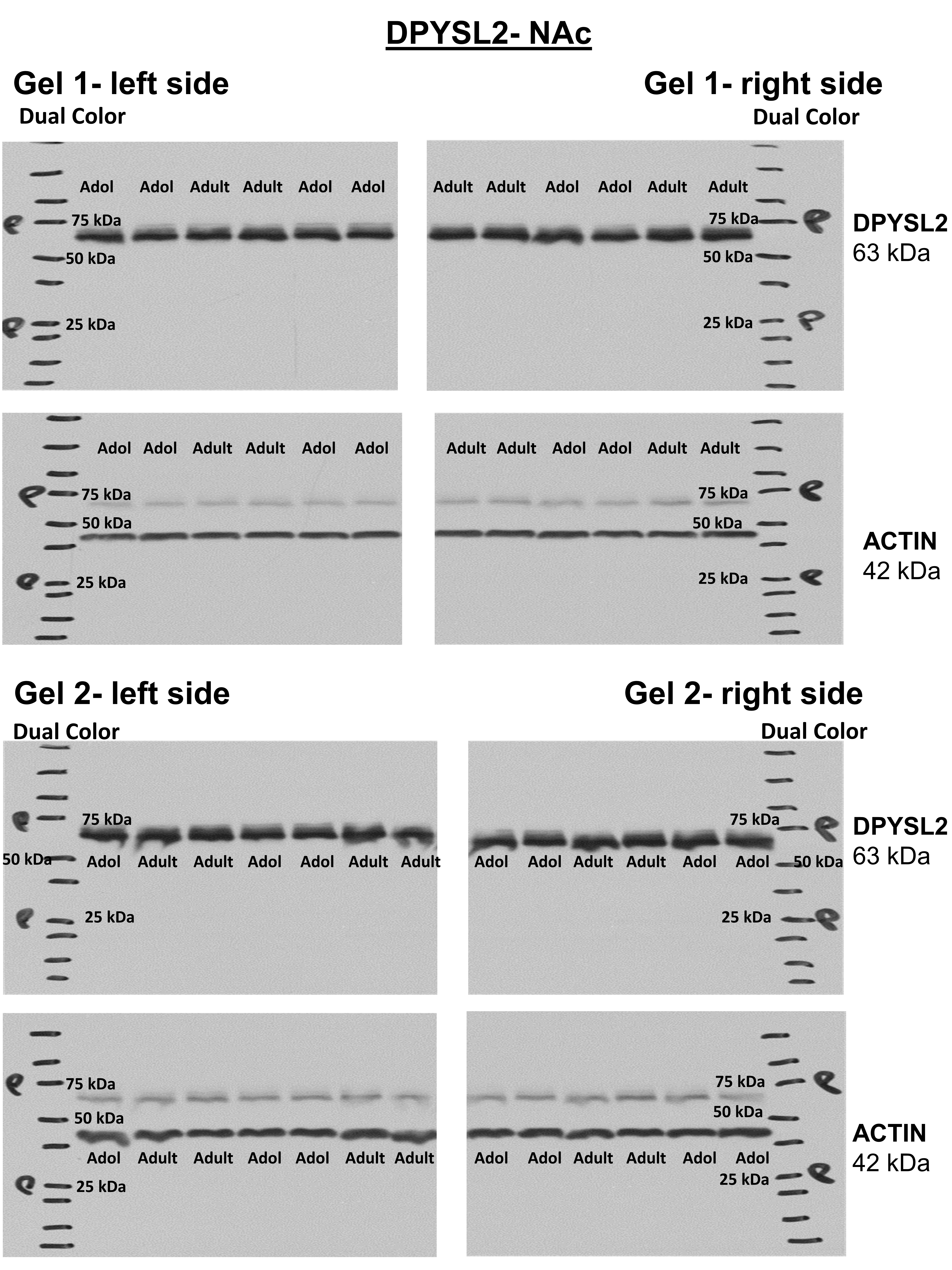

Supplement: S3 Fig — Photos represent the left and right sides of a single 18-lane membrane. (TIF) [file pone.0178391.s003.TIF]

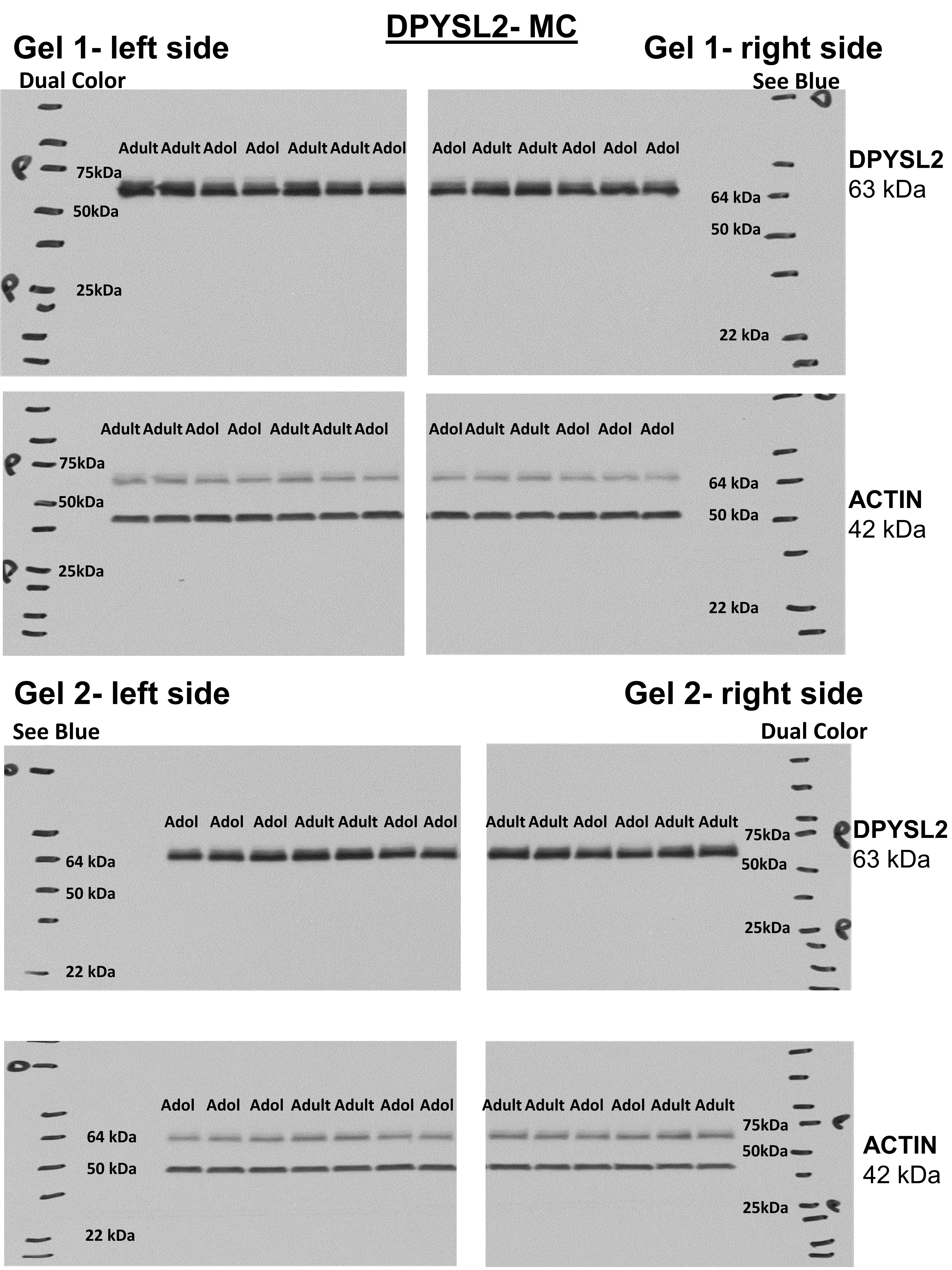

Supplement: S4 Fig — Photos represent the left and right sides of a single 18-lane membrane. (TIF) [file pone.0178391.s004.TIF]

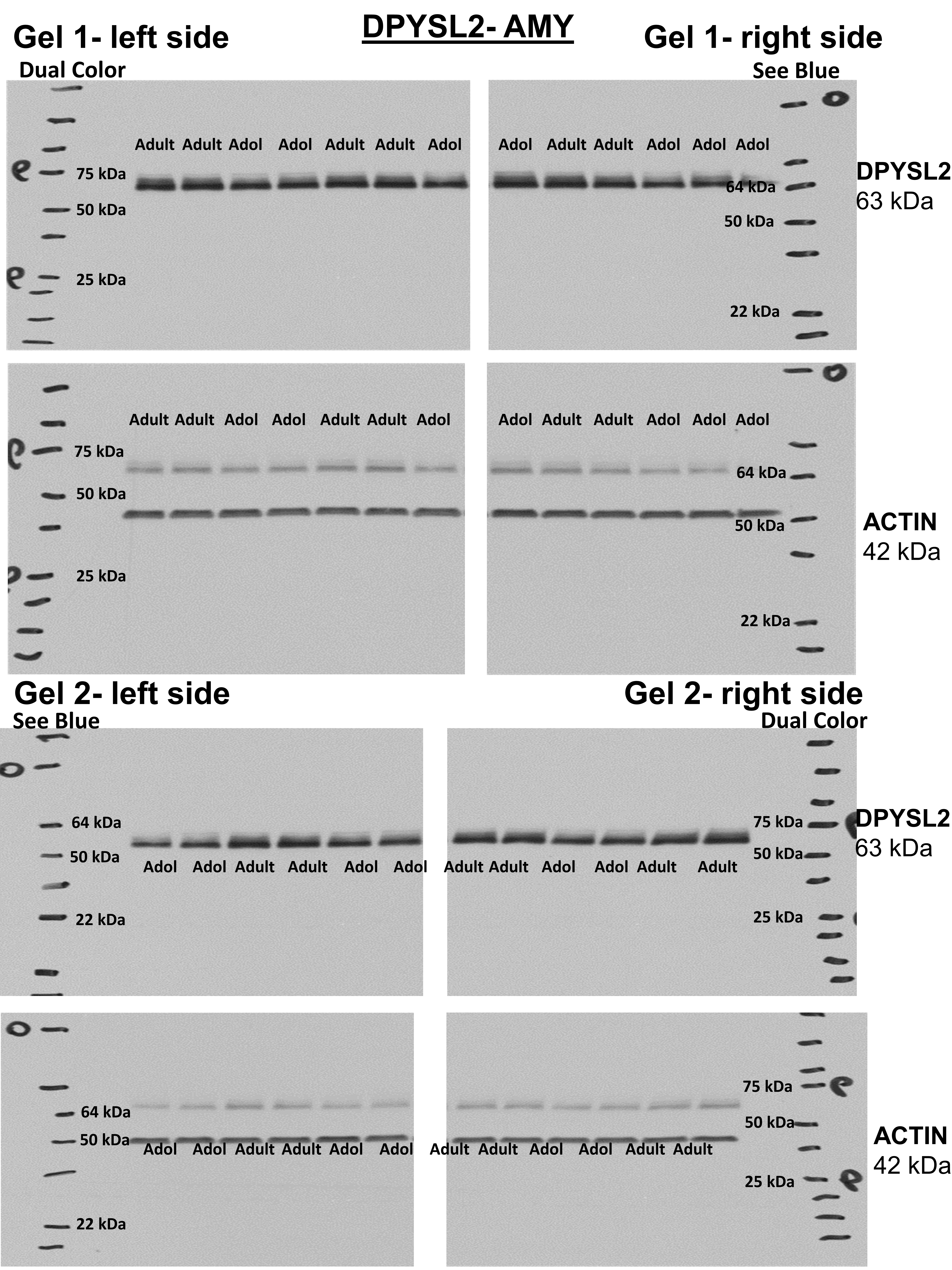

Supplement: S5 Fig — Photos represent the left and right sides of a single 18-lane membrane. (TIF) [file pone.0178391.s005.TIF]

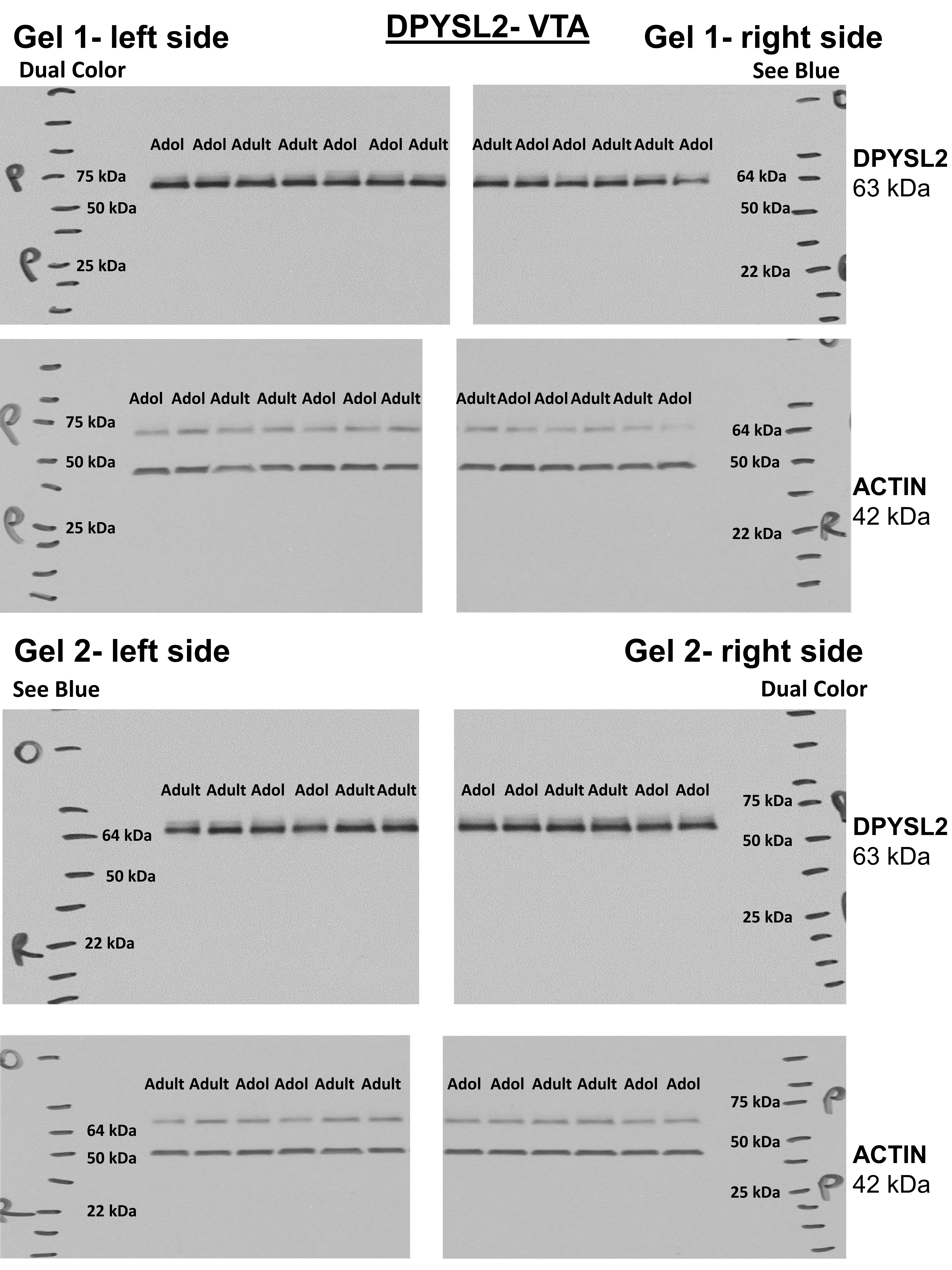

Supplement: S6 Fig — Photos represent the left and right sides of a single 18-lane membrane. (TIF) [file pone.0178391.s006.TIF]

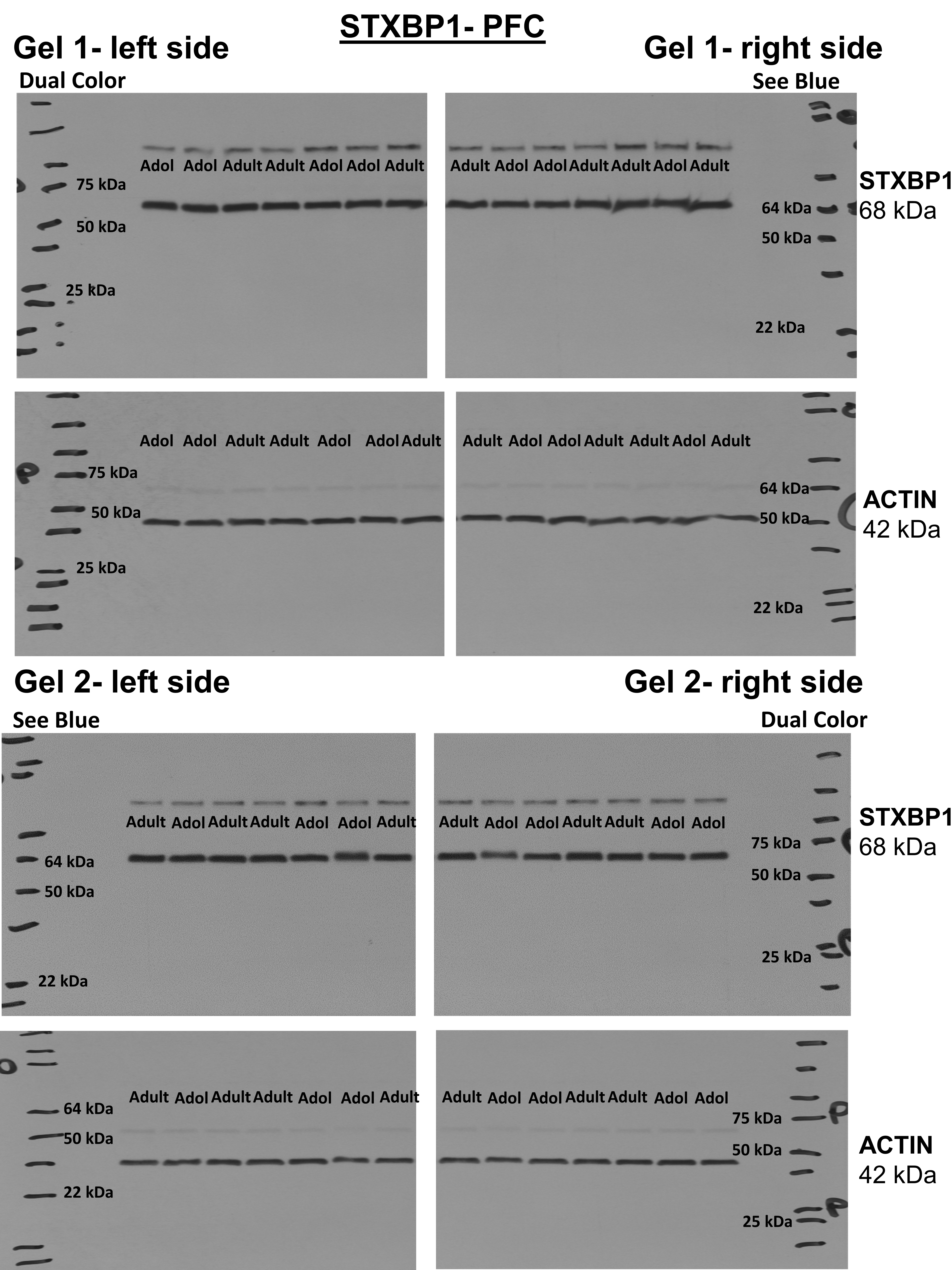

Supplement: S7 Fig — Photos represent the left and right sides of a single 18-lane membrane. Visible bands at ~100 kDa are Drebrin which was probed on the same blot. (TIF) [file pone.0178391.s007.TIF]

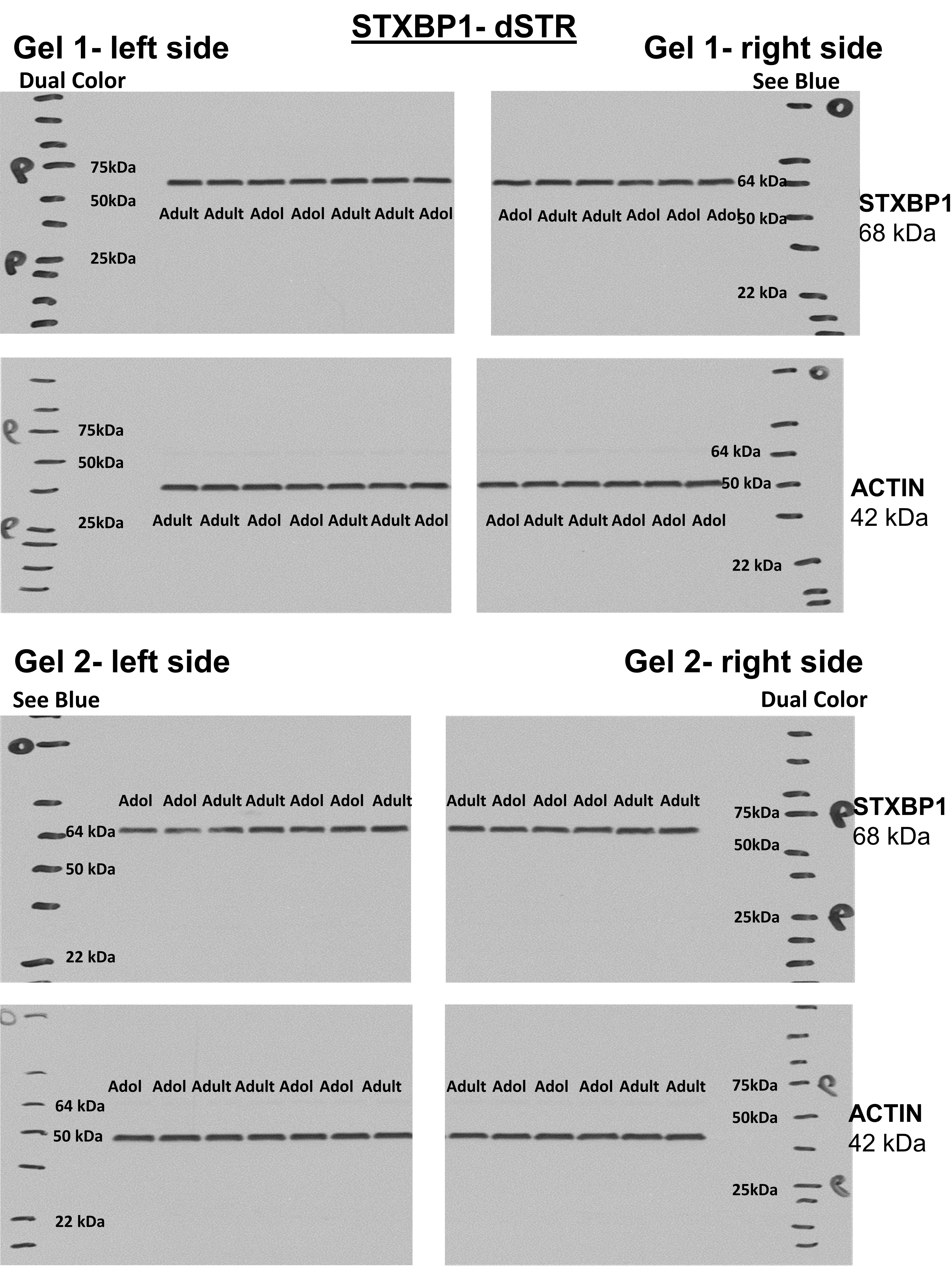

Supplement: S8 Fig — Photos represent the left and right sides of a single 18-lane membrane. (TIF) [file pone.0178391.s008.TIF]

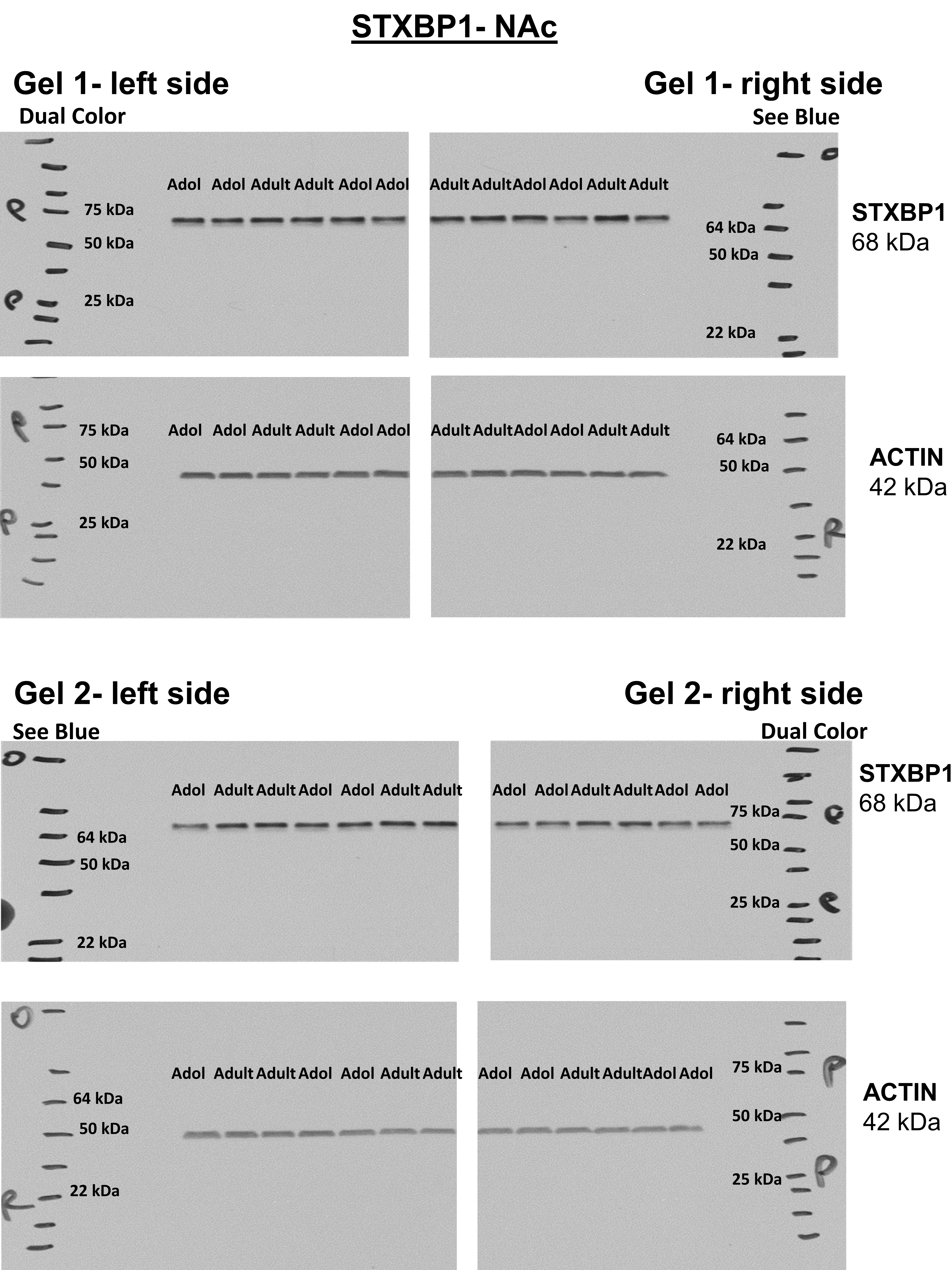

Supplement: S9 Fig — Photos represent the left and right sides of a single 18-lane membrane. (TIF) [file pone.0178391.s009.TIF]

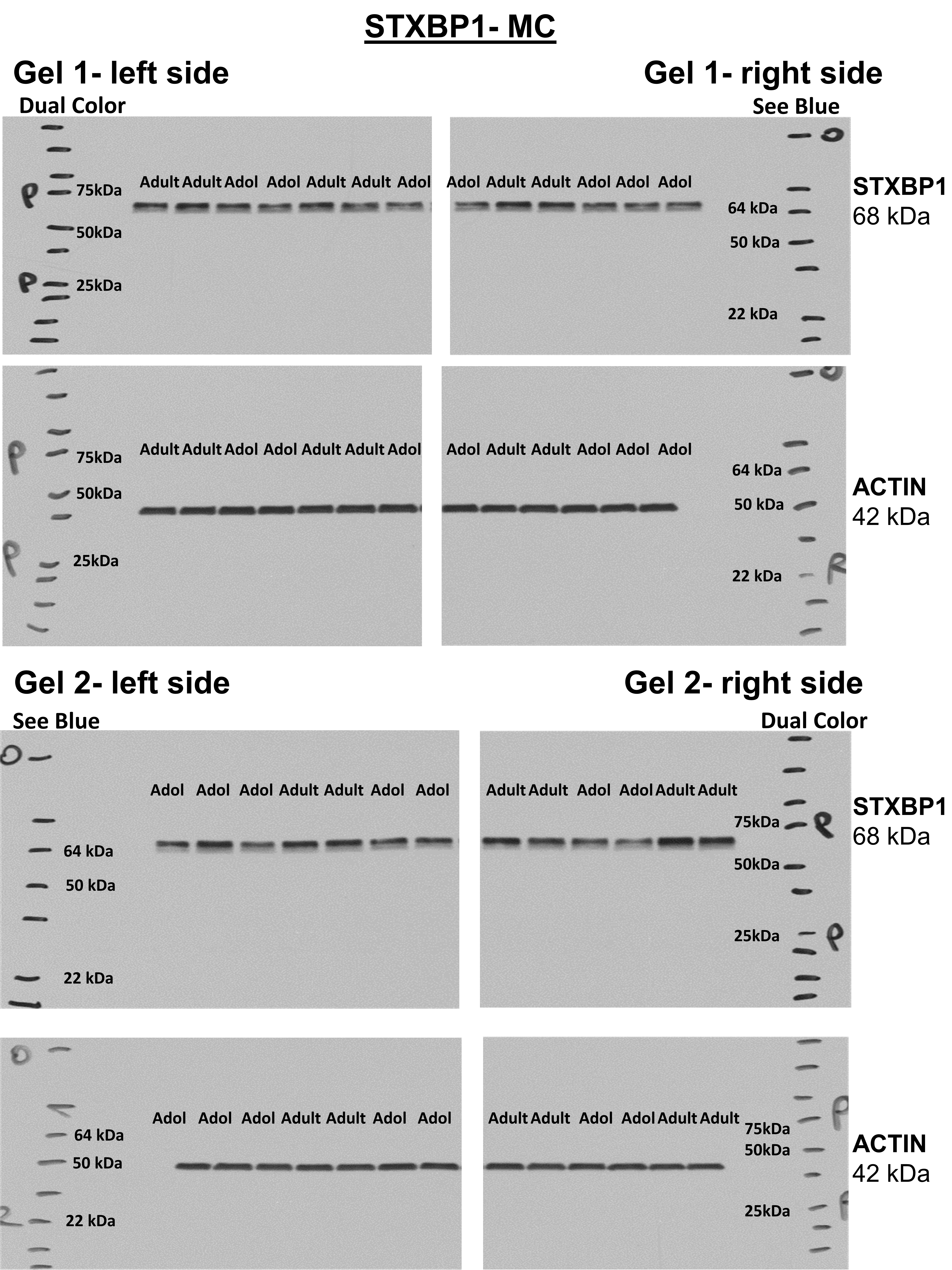

Supplement: S10 Fig — Photos represent the left and right sides of a single 18-lane membrane. (TIF) [file pone.0178391.s010.TIF]

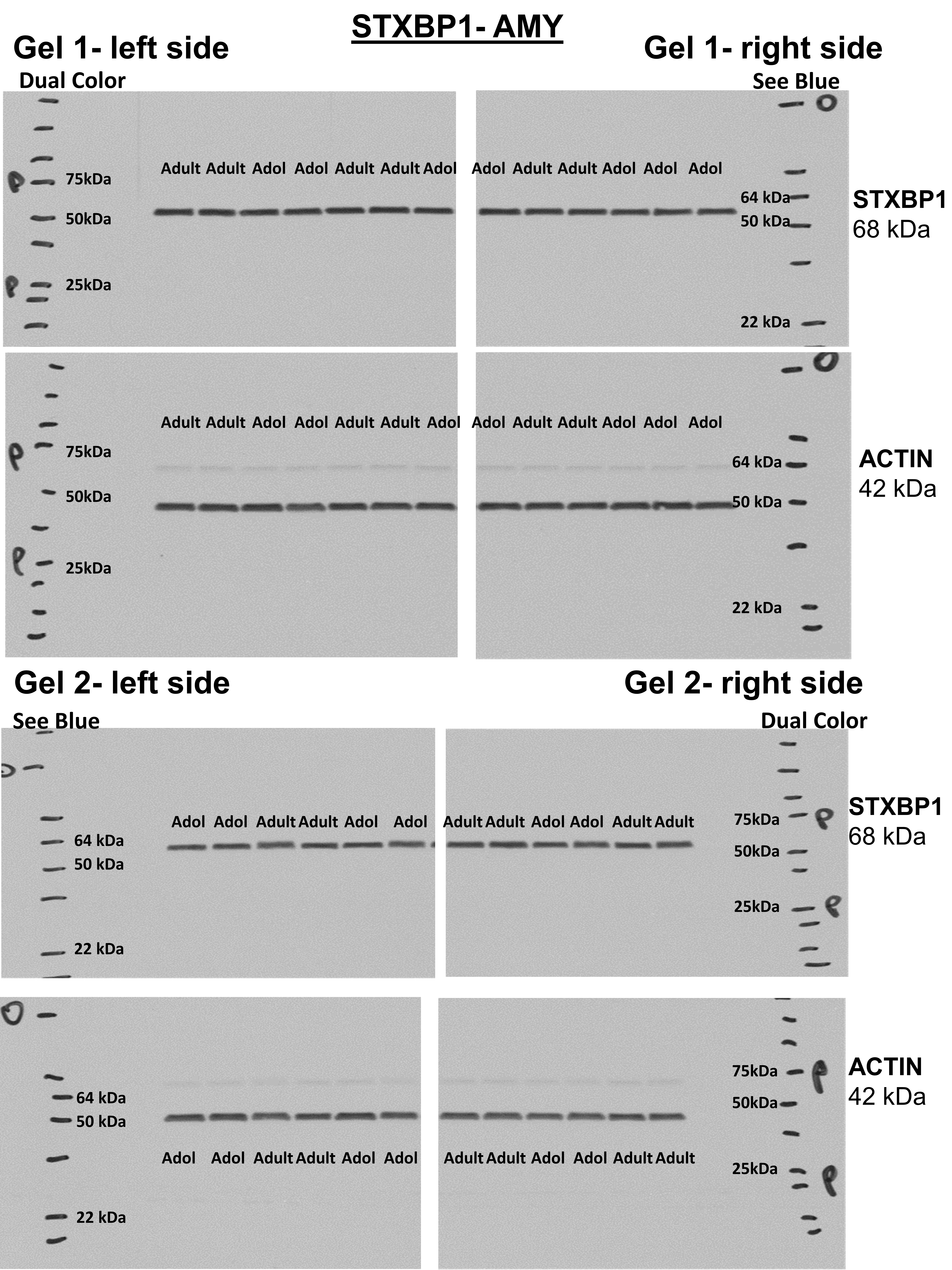

Supplement: S11 Fig — Photos represent the left and right sides of a single 18-lane membrane. (TIF) [file pone.0178391.s011.TIF]

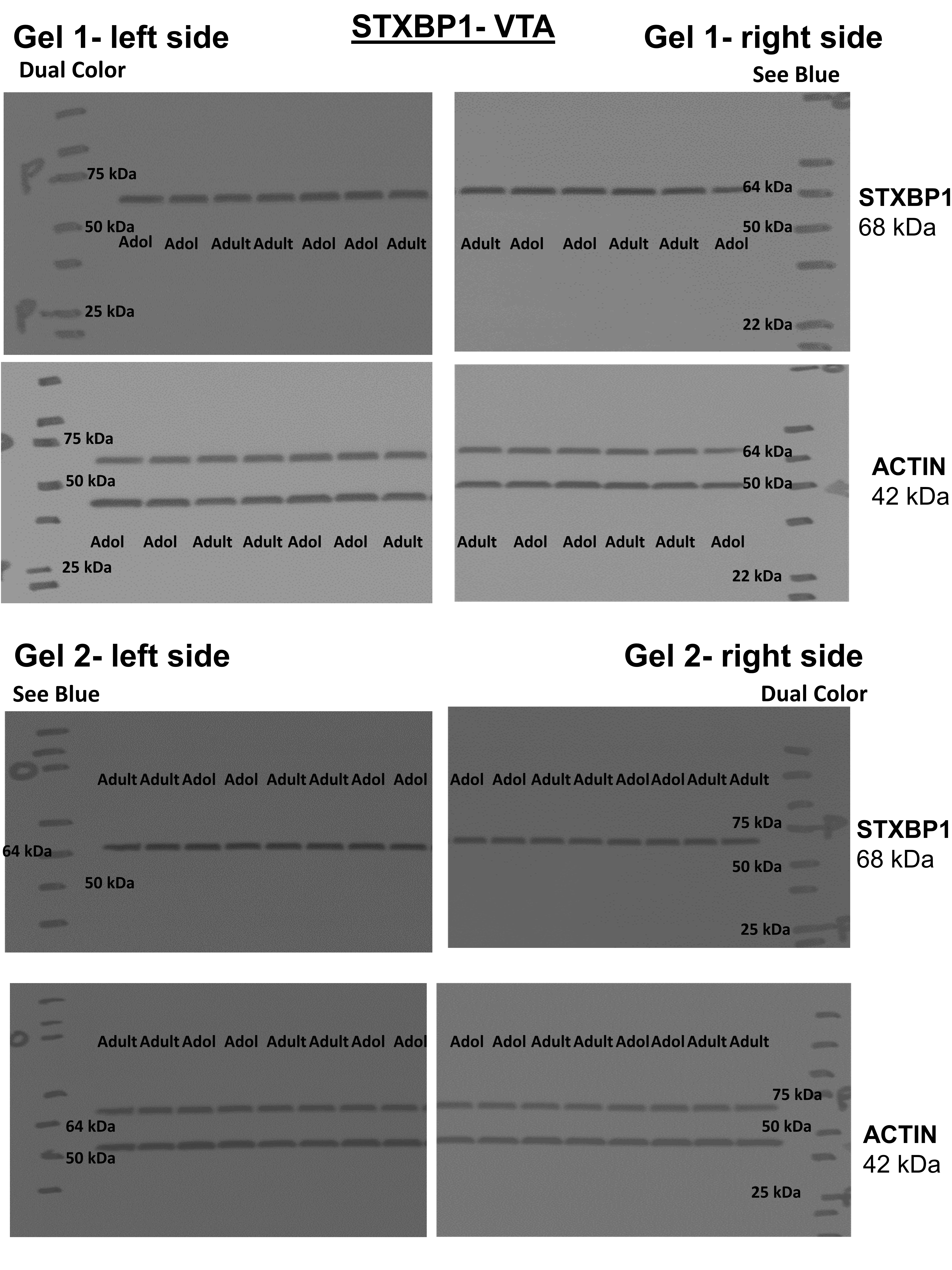

Supplement: S12 Fig — Photos represent the left and right sides of a single 18-lane membrane. (TIF) [file pone.0178391.s012.TIF]

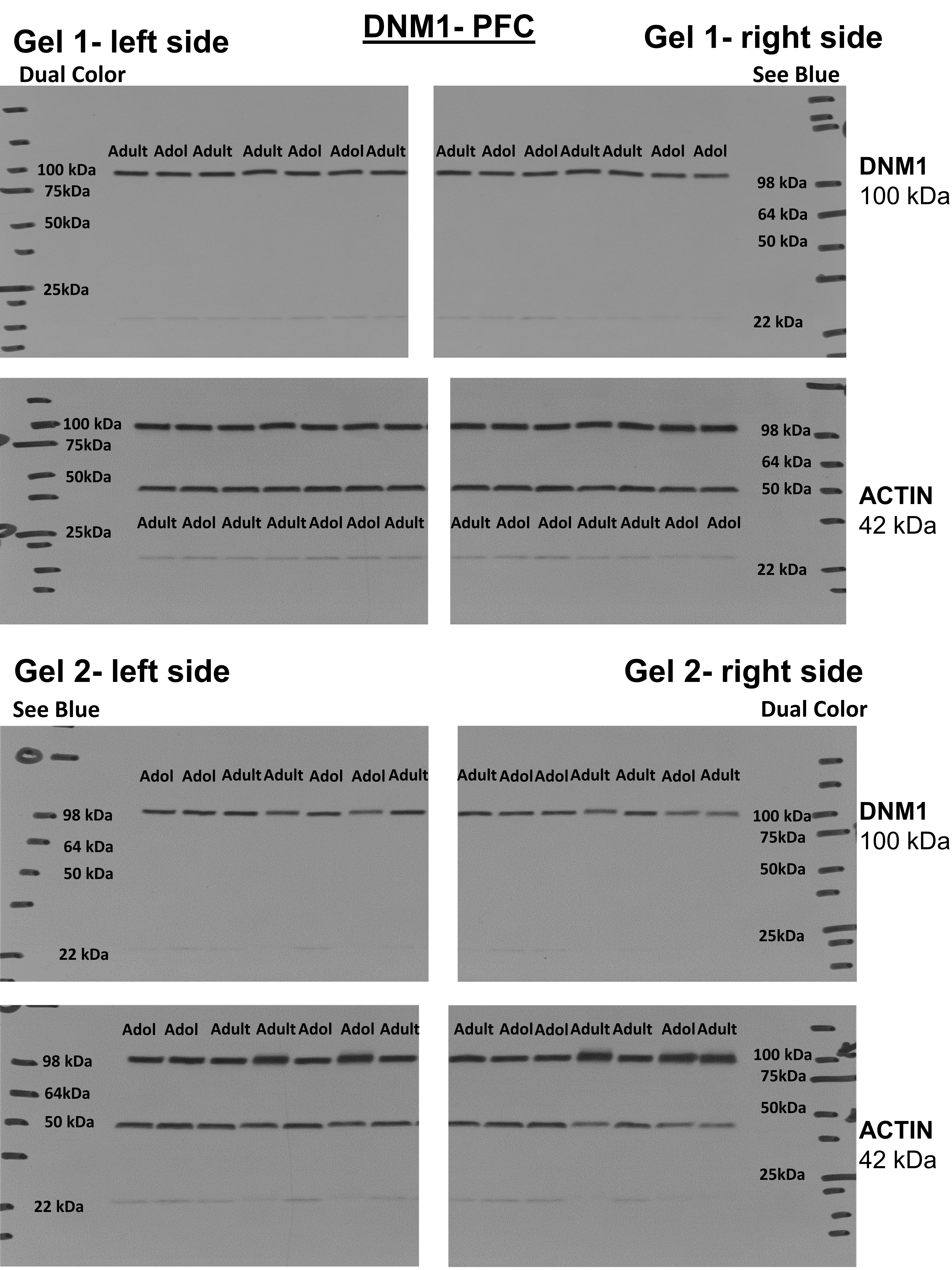

Supplement: S13 Fig — Photos represent the left and right sides of a single 18-lane membrane. Visible bands at ~20 kDa are CFL1 which was probed on the same blot. (TIF) [file pone.0178391.s013.TIF]

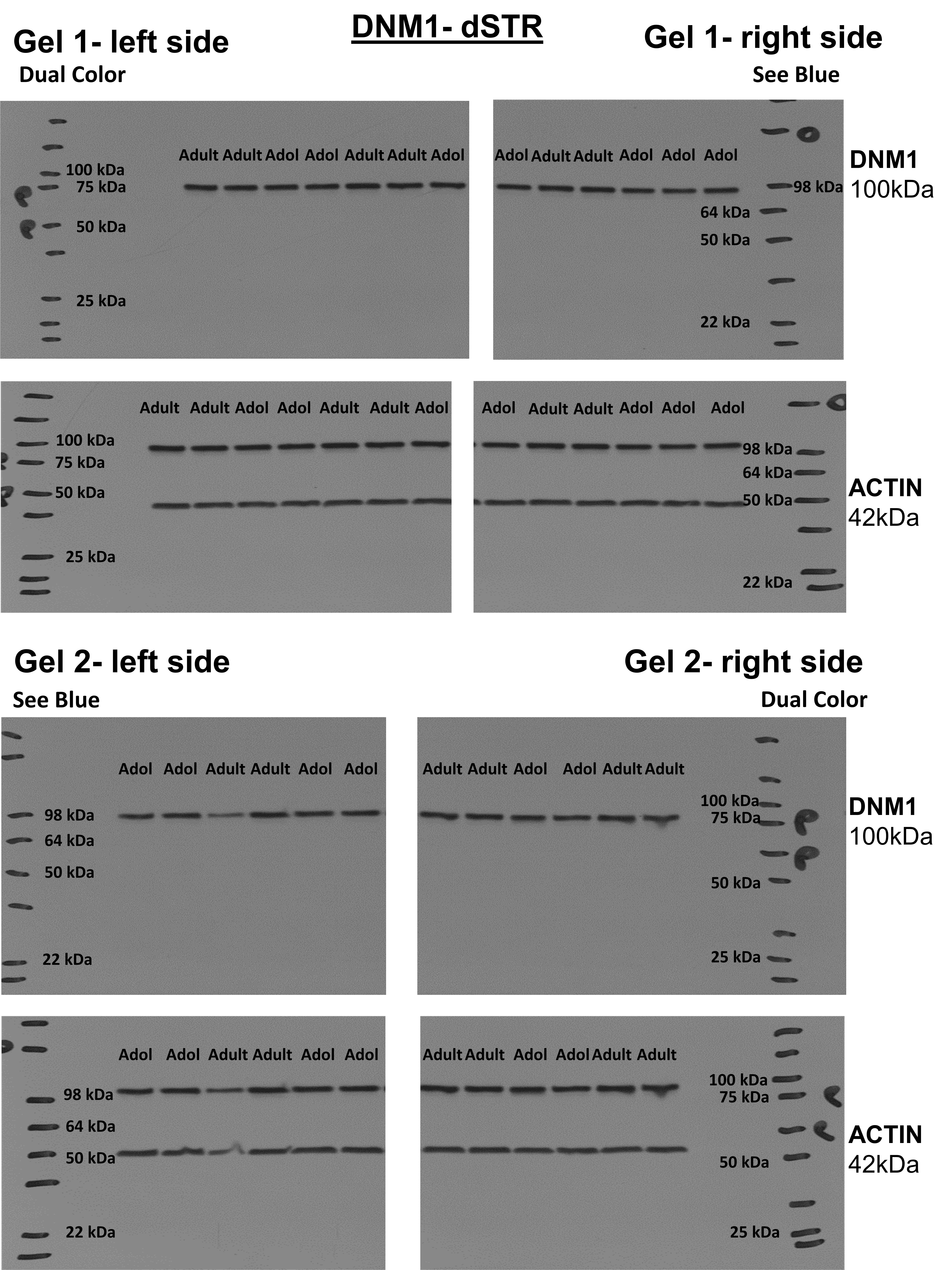

Supplement: S14 Fig — Photos represent the left and right sides of a single 18-lane membrane. Visible bands at ~20 kDa are CFL1 which was probed on the same blot. (TIF) [file pone.0178391.s014.TIF]

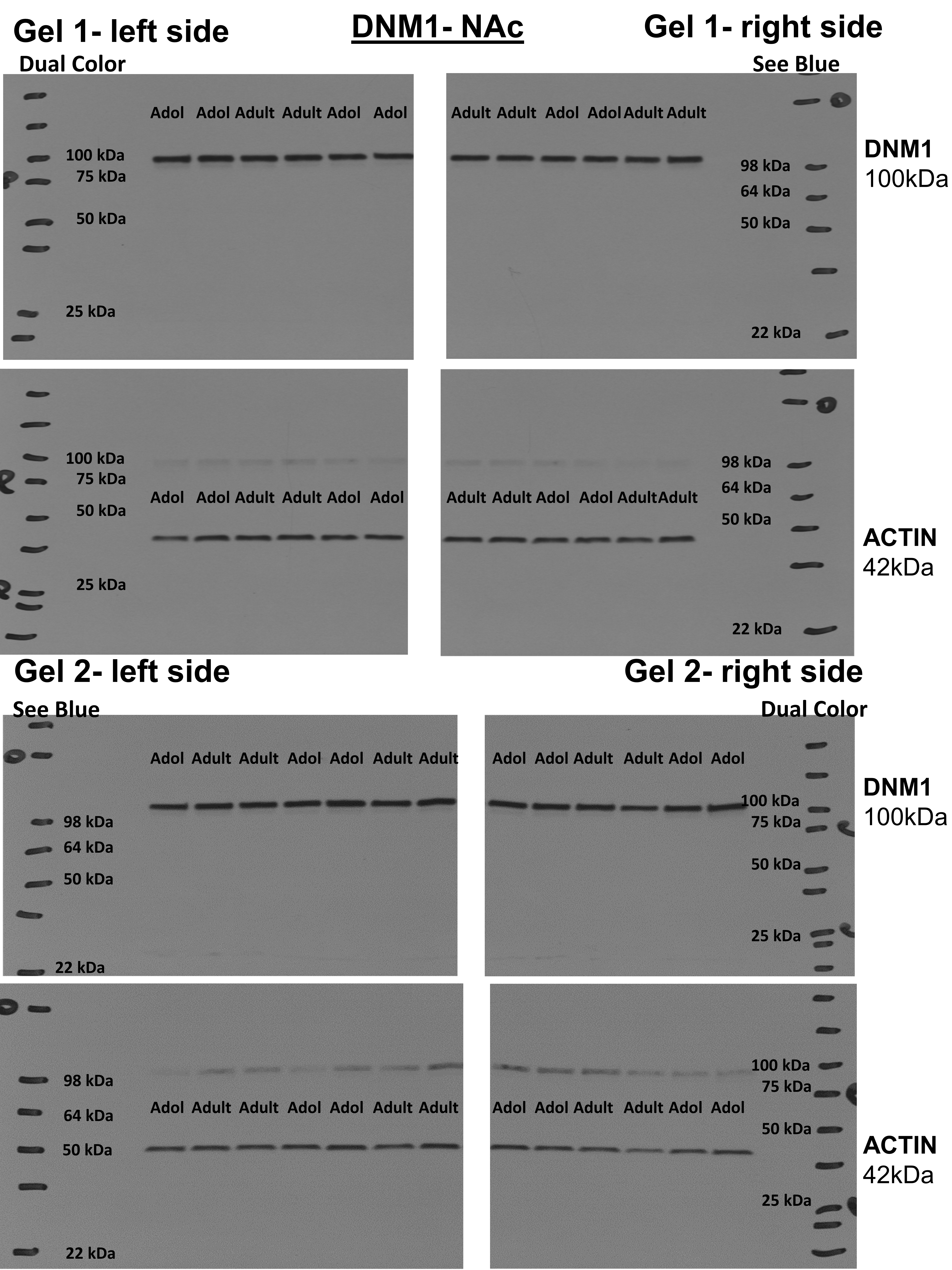

Supplement: S15 Fig — Photos represent the left and right sides of a single 18-lane membrane. Visible bands at ~20 kDa are CFL1 which was probed on the same blot. (TIF) [file pone.0178391.s015.TIF]

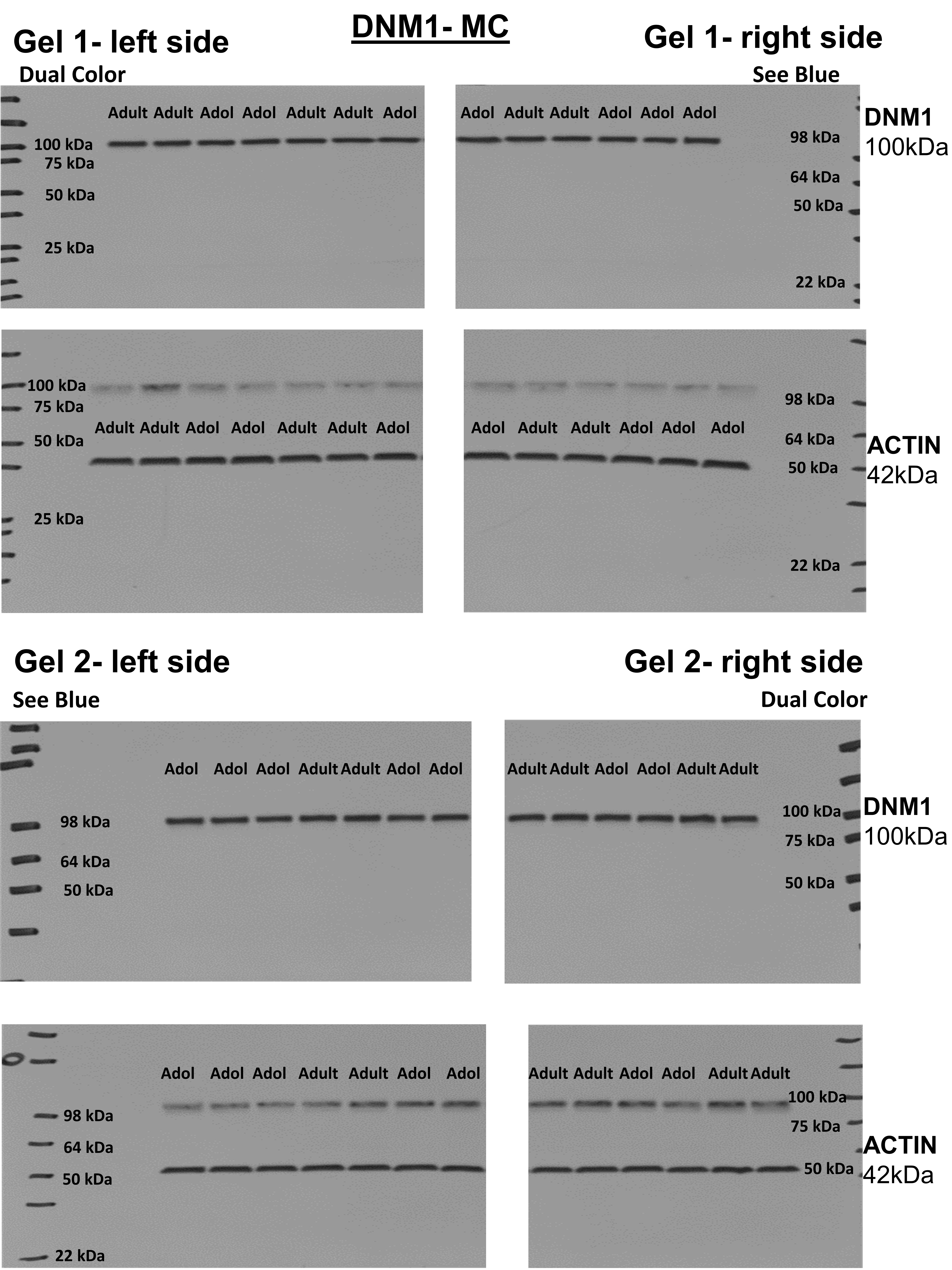

Supplement: S16 Fig — Photos represent the left and right sides of a single 18-lane membrane. Visible bands at ~20 kDa are CFL1 which was probed on the same blot. (TIF) [file pone.0178391.s016.TIF]

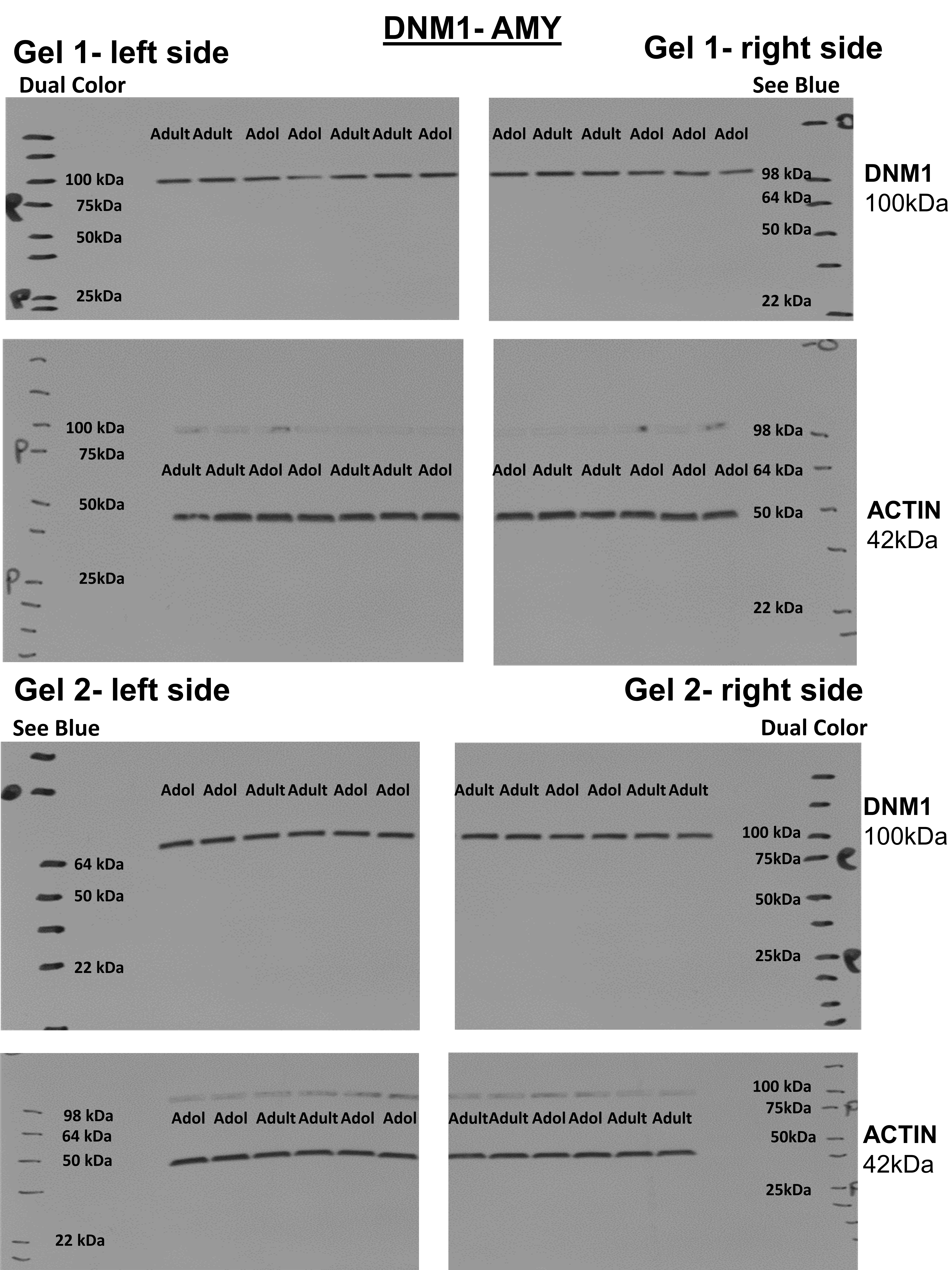

Supplement: S17 Fig — Photos represent the left and right sides of a single 18-lane membrane. Visible bands at ~20 kDa are CFL1 which was probed on the same blot. (TIF) [file pone.0178391.s017.TIF]

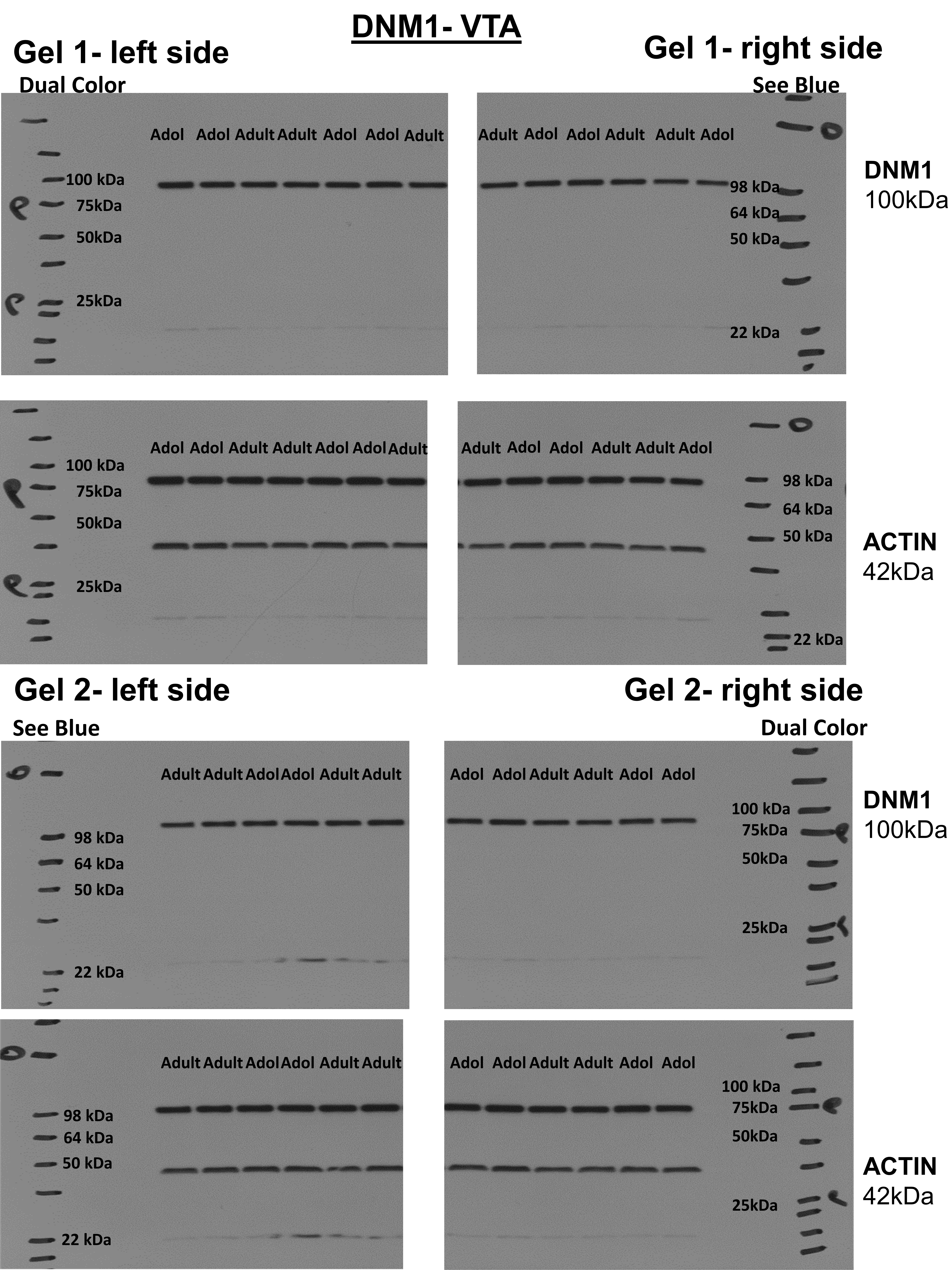

Supplement: S18 Fig — Photos represent the left and right sides of a single 18-lane membrane. Visible bands at ~20 kDa are CFL1 which was probed on the same blot. (TIF) [file pone.0178391.s018.TIF]

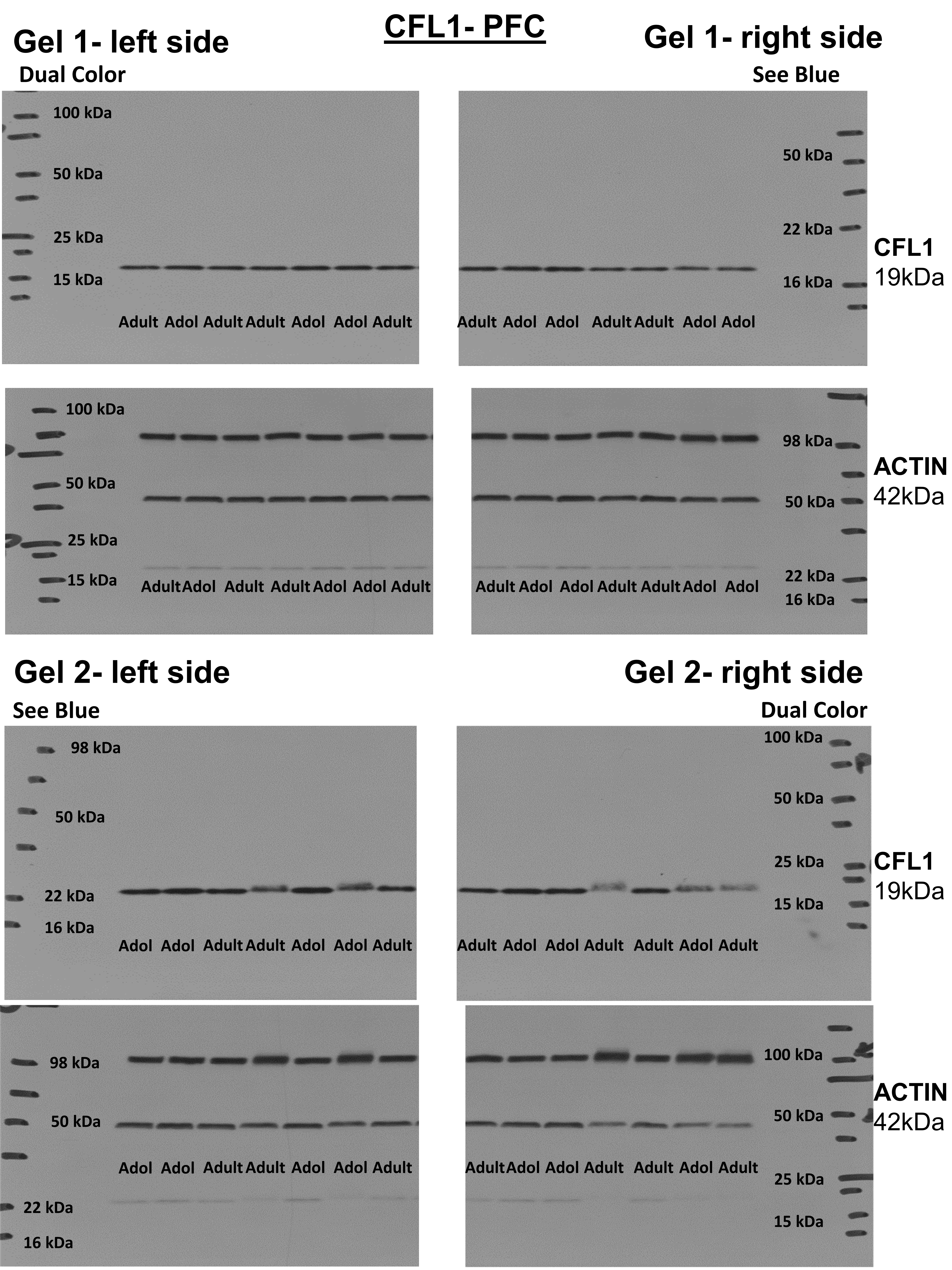

Supplement: S19 Fig — Photos represent the left and right sides of a single 18-lane membrane. Visible bands at ~100kDa are DNM1 which was probed on the same blot. (TIF) [file pone.0178391.s019.TIF]

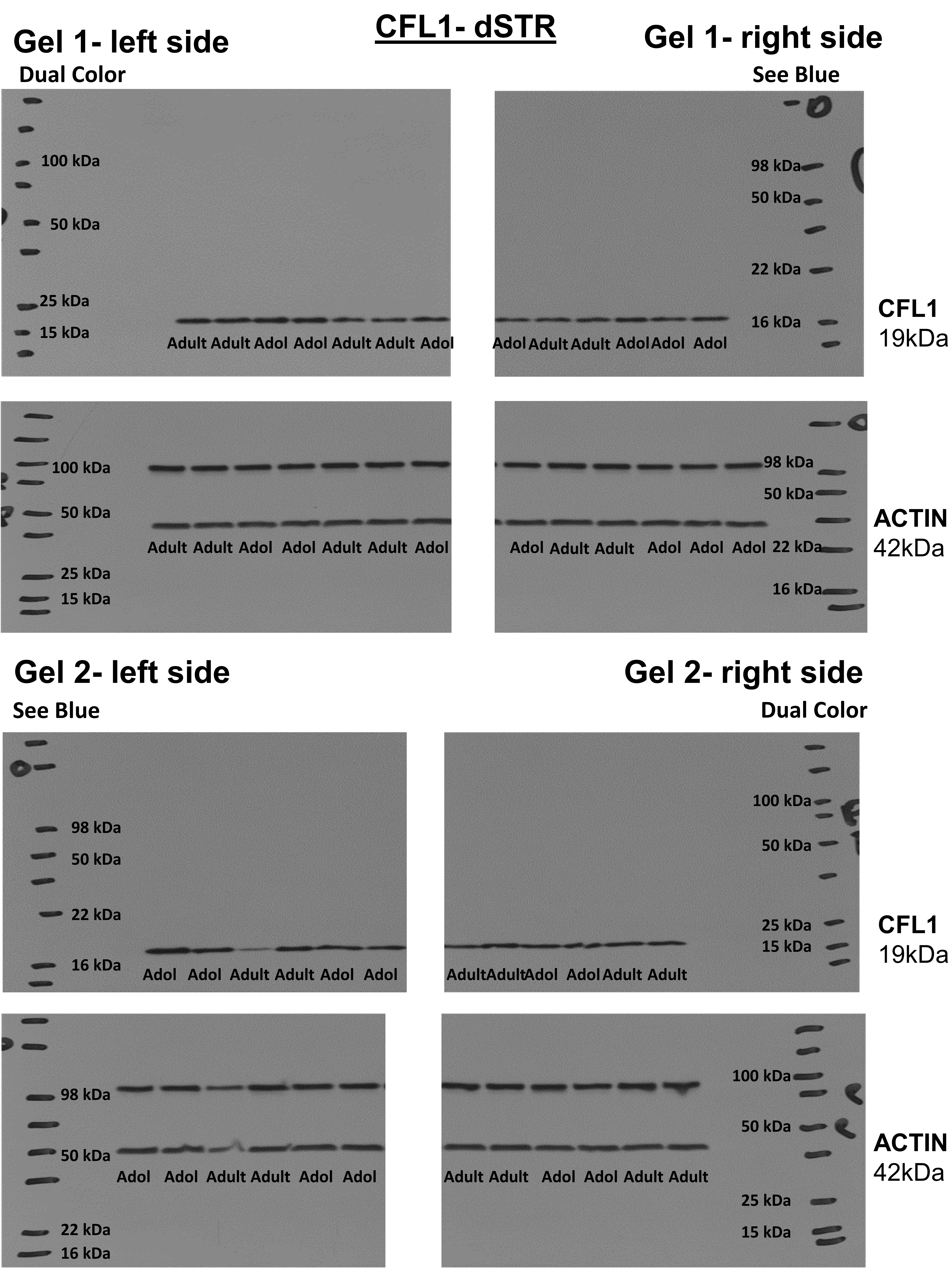

Supplement: S20 Fig — Photos represent the left and right sides of a single 18-lane membrane. Visible bands at ~100kDa are DNM1 which was probed on the same blot. (TIF) [file pone.0178391.s020.TIF]

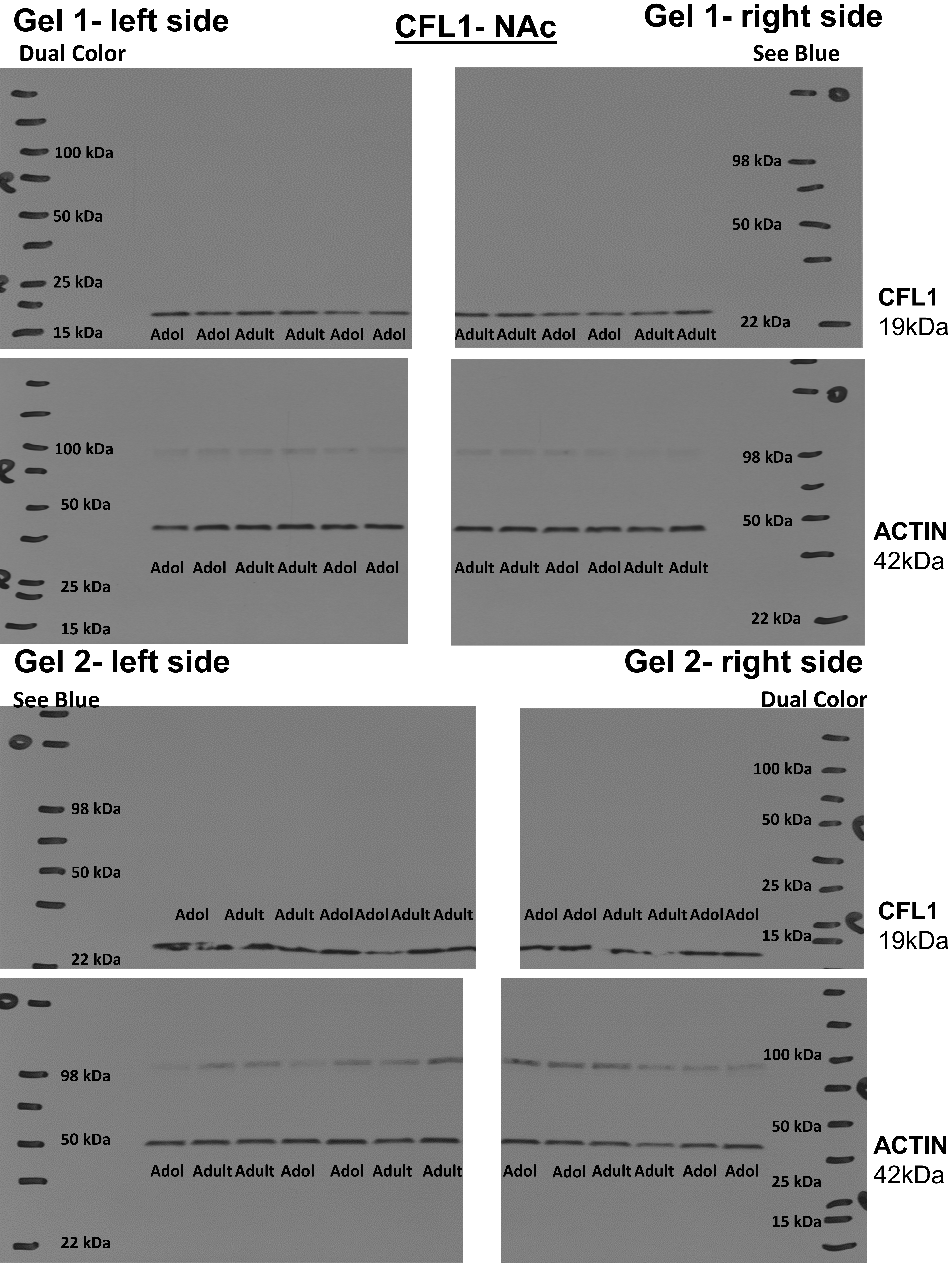

Supplement: S21 Fig — Photos represent the left and right sides of a single 18-lane membrane. Visible bands at ~100kDa are DNM1 which was probed on the same blot. (TIF) [file pone.0178391.s021.TIF]

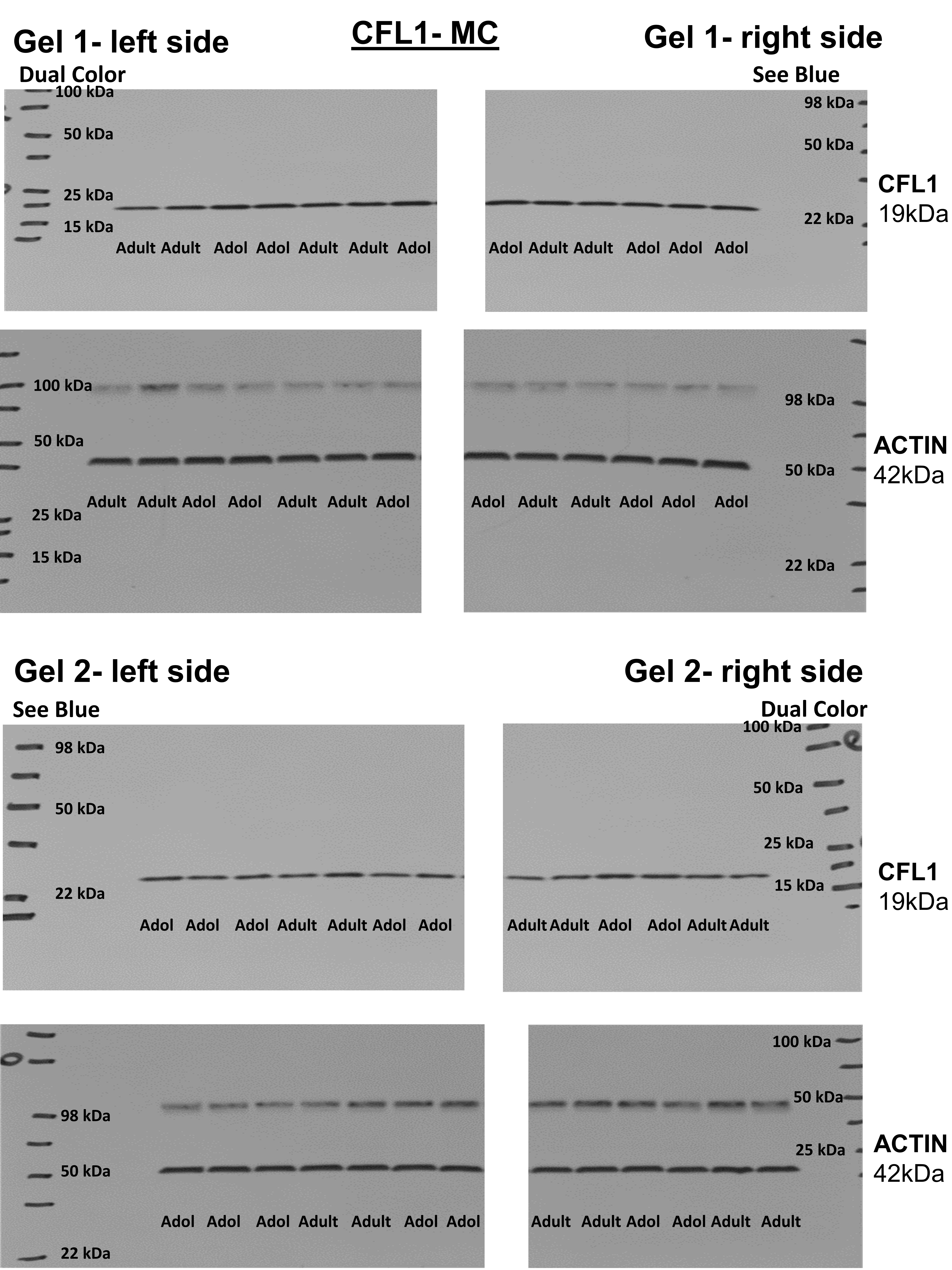

Supplement: S22 Fig — Photos represent the left and right sides of a single 18-lane membrane. Visible bands at ~100kDa are DNM1 which was probed on the same blot. (TIF) [file pone.0178391.s022.TIF]

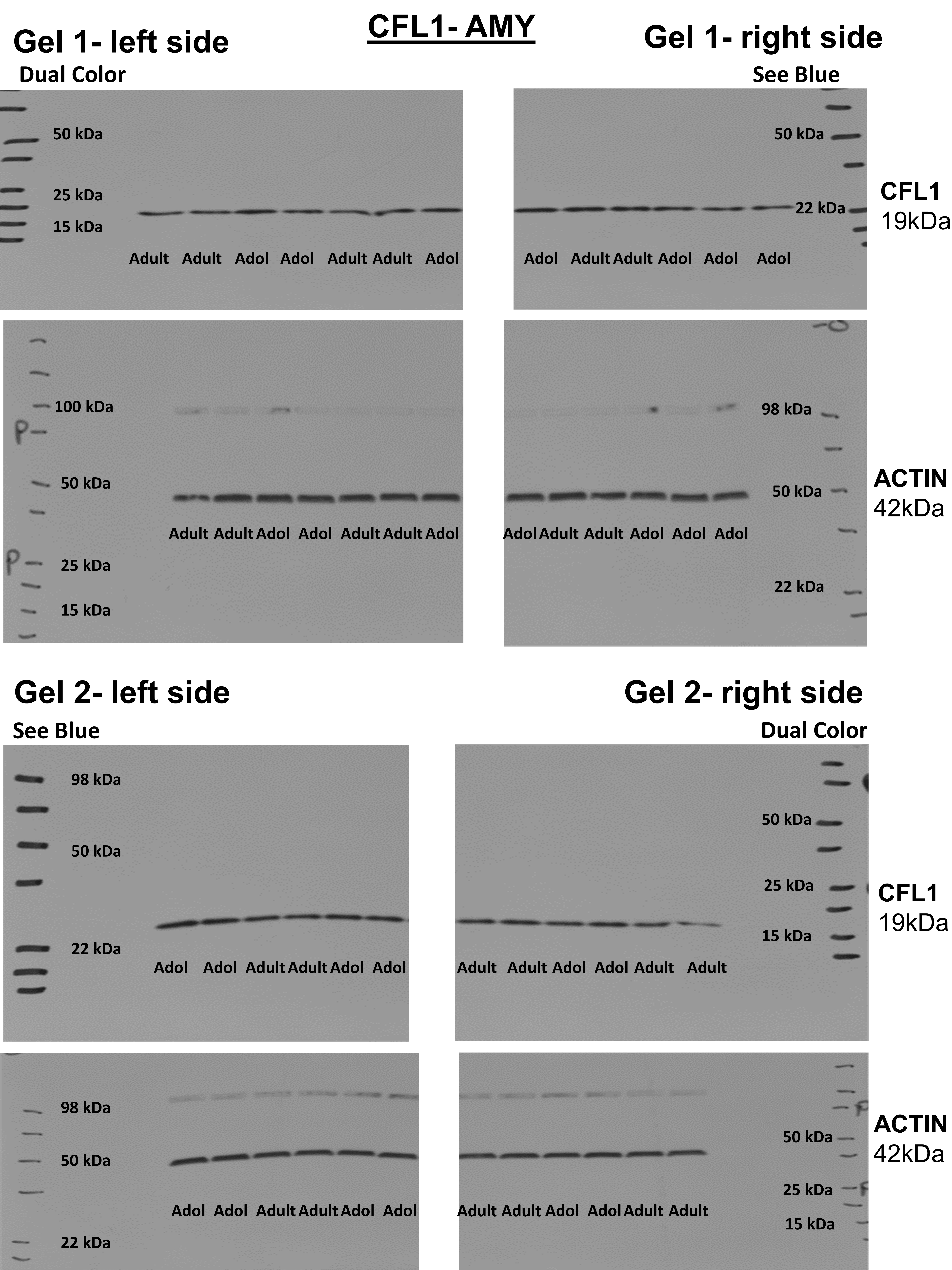

Supplement: S23 Fig — Photos represent the left and right sides of a single 18-lane membrane. Visible bands at ~100kDa are DNM1 which was probed on the same blot. (TIF) [file pone.0178391.s023.TIF]

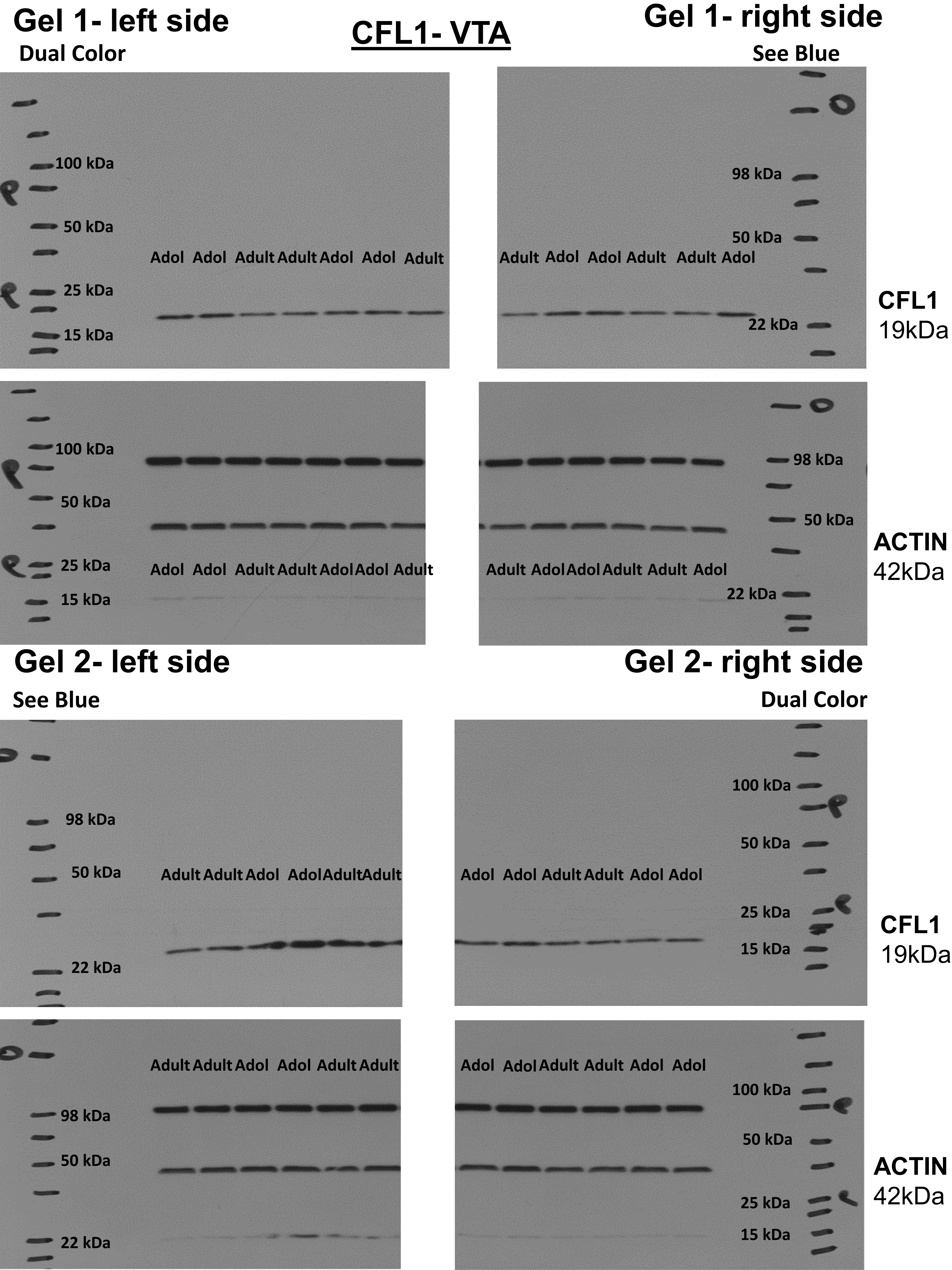

Supplement: S24 Fig — Photos represent the left and right sides of a single 18-lane membrane. Visible bands at ~100kDa are DNM1 which was probed on the same blot. (TIF) [file pone.0178391.s024.TIF]
